# Comparative genomic analysis of *Bacillus atrophaeus* HAB-5 reveals genes associated with antimicrobial and plant growth-promoting activities

**DOI:** 10.3389/fmicb.2024.1384691

**Published:** 2024-06-26

**Authors:** Ghulam Yaseen Dahar, Huan Wei Wang, Faheem Uddin Rajer, Pengfie Jin, Peidong Xu, Manzoor Ali Abro, Abdul Sattar Qureshi, Asad Karim, Weiguo Miao

**Affiliations:** ^1^Key Laboratory of Green Prevention and Control of Tropical Plant Disease and Pests, Ministry of Education, College of Plant Protection, Hainan University Haikou, Haikou, China; ^2^Department of Plant Pathology, Faculty of Crop Protection, Sindh Agriculture University, Tando Jam, Pakistan; ^3^Institute of Biotechnology and Genetic Engineering, University of Sindh, Jamshoro, Pakistan; ^4^Jamil-Ur-Rahman Center for Genome Research, Dr. Panjwani Center for Molecular Medicine and Drug Research, International Center for Chemical and Biological Sciences, University of Karachi, Karachi, Pakistan

**Keywords:** *Bacillus atrophaeus*, whole genome sequencing (WGS), phylogenetic analysis, comparative genome analysis, gene clusters, broad spectrum

## Abstract

*Bacillus atrophaeus* HAB-5 is a plant growth-promoting rhizobacterium (PGPR) that exhibits several biotechnological traits, such as enhancing plant growth, colonizing the rhizosphere, and engaging in biocontrol activities. In this study, we conducted whole-genome sequencing of *B. atrophaeus* HAB-5 using the single-molecule real-time (SMRT) sequencing platform by Pacific Biosciences (PacBio; United States), which has a circular chromosome with a total length of 4,083,597 bp and a G + C content of 44.21%. The comparative genomic analysis of *B. atrophaeus* HAB-5 with other strains, *Bacillus amyloliquefaciens* DSM7, *B. atrophaeus* SRCM101359, *Bacillus velezensis* FZB42, *B. velezensis* HAB-2, and *Bacillus subtilis* 168, revealed that these strains share 2,465 CDSs, while 599 CDSs are exclusive to the *B. atrophaeus* HAB-5 strain. Many gene clusters in the *B. atrophaeus* HAB-5 genome are associated with the production of antimicrobial lipopeptides and polypeptides. These gene clusters comprise distinct enzymes that encode three NRPs, two Transat-Pks, one terpene, one lanthipeptide, one T3PKS, one Ripp, and one thiopeptide. In addition to the likely IAA-producing genes (*trpA, trpB, trpC, trpD, trpE, trpS, ywkB, miaA,* and *nadE*), there are probable genes that produce volatile chemicals (*acoA, acoB, acoR, acuB,* and *acuC*). Moreover, HAB-5 contained genes linked to iron transportation (*fbpA, fetB, feuC, feuB, feuA,* and *fecD*), sulfur metabolism (*cysC, sat, cysK, cysS,* and *sulP*), phosphorus solubilization (*ispH, pstA, pstC, pstS, pstB*, *gltP,* and *phoH*), and nitrogen fixation (*nif3-like, gltP, gltX, glnR, glnA, nadR, nirB, nirD, nasD, narl, narH, narJ,* and *nark*). In conclusion, this study provides a comprehensive genomic analysis of *B. atrophaeus* HAB-5, pinpointing the genes and genomic regions linked to the antimicrobial properties of the strain. These findings advance our knowledge of the genetic basis of the antimicrobial properties of *B. atrophaeus* and imply that HAB-5 may employ a variety of commercial biopesticides and biofertilizers as a substitute strategy to increase agricultural output and manage a variety of plant diseases.

## Introduction

Plant pathogenic fungi, bacteria, viruses, and viroids can reduce agricultural productivity and result in yield losses of up to 14% for various crops (Peng et al., 2021). An essential step in the production of agricultural products is the application of pesticides to combat plant diseases. Chemical pesticides have been used extensively to control plant diseases; without them, the production of fruits, vegetables, and grains would have dropped by 78%, 54%, and 32%, respectively. Regardless of all the above, excessive pesticide usage in agriculture pollutes the environment and has a negative impact on human health (Syed Ab Rahman et al., 2018; Tudi et al., 2021). Furthermore, pesticides can alter the composition of plant-associated microbial communities (Meena et al., 2020). Therefore, the development of ecofriendly pesticide alternatives is urgently required. Due to their safe and environmentally friendly effects on crops, antagonistic bacteria have become a potent substitute for conventional pesticides in the management of crop diseases in recent years. Numerous strains of the *Bacillus* species are being evaluated for use as biopesticides and have gained prominence as a biocontrol agent for plant diseases. *Bacillus* strains are the most promising group of plant growth-promoting rhizobacteria (PGPR), which play important roles in promoting plant growth, enhancing growth hormones, producing antioxidant enzymes, nitrogen fixation, phosphate solubilization, phytohormones, and volatile organic compounds (VOCs), and triggering induced systemic resistance (ISR) by producing different types of secondary metabolites that can potentially inhibit the growth of plant pathogens and control soil-borne diseases (Ongena and Jacques, 2008; Chowdhury et al., 2015; García-Fraile et al., 2015; Fan et al., 2018; Chandran et al., 2020; Samaras et al., 2021).

*Bacillus atrophaeus* is a significant member of the plant growth-promoting rhizobacteria (PGPR), which are known for enhancing plant growth development as well as controlling plant pathogenic fungi and bacteria. When applied to seedlings and harvested fruits, they stimulate plant growth and improve the resistance of plants against insect pests and plant diseases such as powdery mildew and tomato gray mold (Reva et al., 2013; Zhang et al., 2013; Huang et al., 2015). Bacterial secondary metabolites are important not only for producer cells but also have a positive impact on their host. These secondary metabolites have significant applications in agriculture and pharmaceuticals as bioactive compounds (Bornscheuer, 2016). Advances in whole-genome sequencing technology have enabled the detection of putative antimicrobial and genome mining tools, enabling researchers to uncover the molecular basis of strain-versatile lifestyles and prioritize industrially important secondary metabolites at the genomic level. These specialized metabolites represent a possible way to improve crop yield and produce antimicrobial activities to control plant diseases (Chun et al., 2017, 2019; Wang et al., 2020; Iqbal et al., 2021). For example, genome mining of *B. atrophaeus* L193 has revealed a non-ribosomal peptide synthetase gene cluster involved in the production of surfactin, fengycin, bacillomycin, and iturin (Rodríguez et al., 2018).

Genome analysis of *B. atrophaeus* GQJK17 revealed eight gene clusters that produce antimicrobial secondary metabolites such as surfactin, fengycin, bacillaene, and bacillibactin (Ma et al., 2018). Surfactin, fengycin, bacillomycin, iturin, bacillaene, and bacillibactin have been reported to have antimicrobial properties (Wang et al., 2024). Bacillibactin has been reported to inhibit the growth and invasion of *Phytophthora capsici* and *Fusarium oxysporum* (Woo and Kim, 2008; Yu et al., 2011). Surfactin was reported to have a broad spectrum of antibacterial activity to significantly inhibit bacterial diseases such as Arabidopsis root infection caused by *Psuedomonas syringae* and tomato wilt caused by *Ralstonia solanacearum* (Bais et al., 2004; Xiong et al., 2015), and fengycin exhibited antifungal activity against a broad spectrum of filamentous fungi (Vanittanakom et al., 1986).

In this study, we selected the *B. atrophaeus* HAB-5 strain, which was isolated from the cotton rhizosphere in Xinjiang Province, Republic of China. The HAB-5 strain has potential as a biological agent for antifungal and antiviral agents. Previous studies conducted in our laboratory have shown that HAB-5 can provide high control efficacy against 22 plant pathogenic fungi such as *Alternaria alternata* MfAa-1, *Alternaria brassicicola* MfAb-1, *Colletotrichum gloeosporioides* MnCg-2, *Colletotrichum musae* BnCy-1, *Colletotrichum gloeosporioides* MnCg-3, *Colletotrichum gloeosporioides* MnCg-4, *Colletotrichum gloeosporioides* MnCg-1, *Colletotrichum gloeosporioides* MnCg-5, *Colletotrichum gloeosporioides* MnCg-6, *Colletotrichum gloeosporioides* MnCg-7, *Colletotrichum gloeosporioides* MnCg-9, *Colletotrichum gloeosporioides* MnCg-10, *Colletotrichum gloeosporioides* MnCg-11, *Corynespora cassiicola* HbCc-1, *Corynespora cassiicola* HbCc-2, *Curvularia geniculata* CyCg-1, *Fusarium oxysporum* f. sp. cubense FOC-4, *Fusarium proliferatum* PgFp-1, *Fusarium oxysporum* PgFp-2, *Phyllosticta theaefolia*, *Phytophtora nicotianae,* and *Trichothecium roseum* PyTr-1, and the inhibition range from 21.07 to 67.29% was recorded (Rajaofera et al., 2020). It also prevents disease infection, protects tobacco seedlings from *P. nicotianae*, and exhibits an antiviral effect against tobacco mosaic virus (TMV) by activating the signaling of regulatory genes (NPR1), defense genes (PR-1a, PR-1b, and chia5), and hypertensive response-related genes (Hsr 203j and Hin1; Rajaofera et al., 2019). Similarly, HAB-5 was found to be effective against *C. gloeosporioides* through the volatilization of antimicrobial volatile compounds such as octadecane, hexadecanoic acid, methyl ester, and chloroacetic acid, tetradecyl ester, chloroacetic acid, tetradecyl ester, octadecane, hexadecanoic acid, and methyl ester (Rajaofera et al., 2018). In addition, the HAB-5 strain exhibited a remarkable ability to improve the growth of tobacco plants, and the inoculated plant exhibited a significant increase in fresh shoot weight, dry shoot weight, fresh root weight, and dry root weight by 76.47%, 80.58%, 71.71%, and 82.10%, respectively, compared with the non-inoculated control plant (Rajaofera et al., 2019). These interesting features have piqued our interest in *B. atrophaeus* HAB-5. Analysis of the whole genome of HAB-5 revealed the PGPR and biological control activities. The objective of this study was to perform genome assembly of *B. atrophaeus* HAB-5 and comparative genome analysis of *B. atrophaeus* HAB-5 with *B. amyloliquefaciens* DSM7, *B. atrophaeus* SRCM101359, *B. velezensis* FZB42*, B. velezensis* HAB-2, and *B. subtilis* 168. In addition to evaluating gene clusters encoding potential secondary metabolites of *B. atrophaeus*, HAB-5 may contribute to plant growth-promoting and biocontrol activities.

## Materials and methods

### *Bacillus atrophaeus* HAB-5 strain selection and genomic DNA extraction

HAB-5 was isolated from Xinjiang Province, Republic of China (Rajaofera et al., 2019) and preserved in the Key Laboratory of Green Prevention and Control of Tropical Plant Disease and Pest at Hainan University, Ministry of Education. The HAB-5 was cultivated in Luria–Bertani (LB) agar medium at 37°C with shaking at 180 rpm and was used to extract genome DNA. The bacterial culture was subjected to genomic DNA extraction using a commercial kit according to the manufacturer’s instructions (Sigma Aldrich, St. Louis, MO, United States). NanoDrop and Qubit (Thermo Fisher Scientific United States) were utilized for optical density measurement and quality control.

### Genome sequences of *Bacillus atrophaeus* HAB-5

The complete genome of HAB-5 was sequenced by third generation sequencing on the PacBio RS II sequencing platform (Pacific Biosciences). Fragment DNA samples were sheared and treated with Exo VII to remove single-stranded ends, and size selection was performed to retain longer reads (>10 k reads) for sequencing. Blunt reactions were performed, and the SMRT bell template was annealed for sequencing. The large insert libraries were sequenced through single-molecule real-time (SMRT) sequencing, and the cells were run on the Pac Biosciences RS II systems using P6-C6 chemistry. After 180 min of mode data collection, all reads were spliced into contigs and combined into scaffolds.

### Genome assembly, gene function annotation, and genome component prediction of *Bacillus atrophaeus* HAB-5

The Pac Bio reads were assembled into contigs using *de novo* hierarchical genome assembly process (HGAP) software in the single-molecule real-time sequencing (SMRT) portal using default parameters (Chin et al., 2013, 2016). The assembly results were then corrected based on NGS data using Pilon software (Walker et al., 2014). Finally, the gaps between contigs were filled by comparing the contigs assembled from PacBio using MUMmer software (Delcher et al., 1999). The genome sequences were annotated by the National Center for Biotechnology Information (NCBI) by using the Prokaryotic Genomes Automatic Annotation Pipeline (PGAAP). Functional description of putative protein-encoding genes was performed using BLASTx, with an E-value of 1e − 5. We used GenoVi software for circular genome representations.^1^ The Kyoto Encyclopedia of Genes and Genomes (KEGG) orthology assignment and prediction of KEGG pathways were performed by Kanehisa et al. (2004) to identify the components of cellular processes (CP), environmental, genetic, human disease, metabolism, and organismal system pathways. The COG (Cluster of Orthologous Group) annotated the predicted genes in accordance with Tatusov et al. (2003); the gene ontology (GO) was completed by Bard and Winter (2000). Genome components were predicted using a glimmer^2^ using Markov models. Scam-SE was used for tRNA, rRNA, and sRNA recognition, and other types of RNAmmers were predicted by comparison with the Rfam database (Lowe and Eddy, 1997).

### The estimation of the core and pan genomes

To categorize the core and pan genomes, HAB-5 was analyzed using the Prokaryotic Genome Annotation Pipeline (PGAP) to identify core orthologs from strains (Zhao et al., 2012). The size of the core genome was determined as the number of common genes shared by all analyzed genomes, and the pan-genome size was defined as the sum of all gene families. Species-specific core orthologous genes and strain-specific unique genes were also examined in the HAB-5 genome sequences. The Ortho MC tool was used to identify core-specific genes in the genomes (Li et al., 2003). The gene accumulation curve was produced using the R package gg plot 2 using the results from Roary.

### Gene family clustering of *Bacillus atrophaeus* HAB-5 and collinearity analysis

Hclustersg software was used to carry out gene family alignment (Maqbool and Babri, 2007), and muscle software was used to analyze the alignment sequence for the cluster gene family (Edgar, 2004). The parameters were set as follows: a Blastp E-value threshold of 1e-5 to ensure the quality of the comparisons. Genome-wide collinearity among strains HAB-5, DSM7, SRCM101359, FZB42, HAB-2, and 168 was determined using the BLASTp database, with an e-value of ≤1e − 5 and an identity threshold of ≥85% at both nucleic acid and amino acid levels. For the analysis of genome synteny and collinearity, D-GENIES and C-Sibelia software were used. Visualization of the alignment of the synteny blocks was achieved using Circos (Krzywinski et al., 2009; Minkin et al., 2013; Cabanettes and Klopp, 2018).

### Phylogenetic trees and heat map synteny

All genomes used in this study were downloaded in the FASTA format from the NCBI database to construct neighbor-joining phylogenetic trees based on 16Sr RNA. Molecular Evolutionary Genetic Analysis (MEGA) was used to construct neighbor-joining phylogenetic trees (Tamura et al., 2011) with the p-distance model and 1,000 bootstraps. An analysis subset of SNPs identified in all single-copy genes. A phylogenetic tree of SNPs was constructed using HAB-5 against the reference genomes by Tree Best (Nandi et al., 2010) with 1,000 bootstraps. The average nucleotide identity analysis was performed using Jsspecies 1.2.1 (Richter et al., 2016). The CIMminer^3^ was used for heat maps based on the Average Nucleotide Identity values, and pairwise genome alignment for synteny was performed using Mauva Version 2.4.0 (Darling et al., 2004).

### Genome mining analysis of secondary metabolite gene clusters

The web-based tool, Antibiotics and Secondary Metabolites Analysis SHell (antiSMASH 7.0) software, was used to predict the biosynthesis gene clusters of secondary metabolites in HAB-5 (Blin et al., 2023). antiSMASH is available at http://antismash.secondarymetabolites.org/. The assembled sequences were uploaded to antiSMASH reports containing both known and unknown clusters to identify similar clusters by genome comparisons with detailed NRP function annotation, and the chemical structure of the gene cluster was generated (Medema et al., 2011; Blin et al., 2013; Weber et al., 2015). The Roary pan genome pipeline was used to identify gene cluster homologies, and gene cluster synteny maps were produced using the R package genoPlotR (Guy et al., 2010; Page et al., 2015).

## Results

### Genome sequences and features of *Bacillus atrophaeus* HAB-5

**Figure 1:**
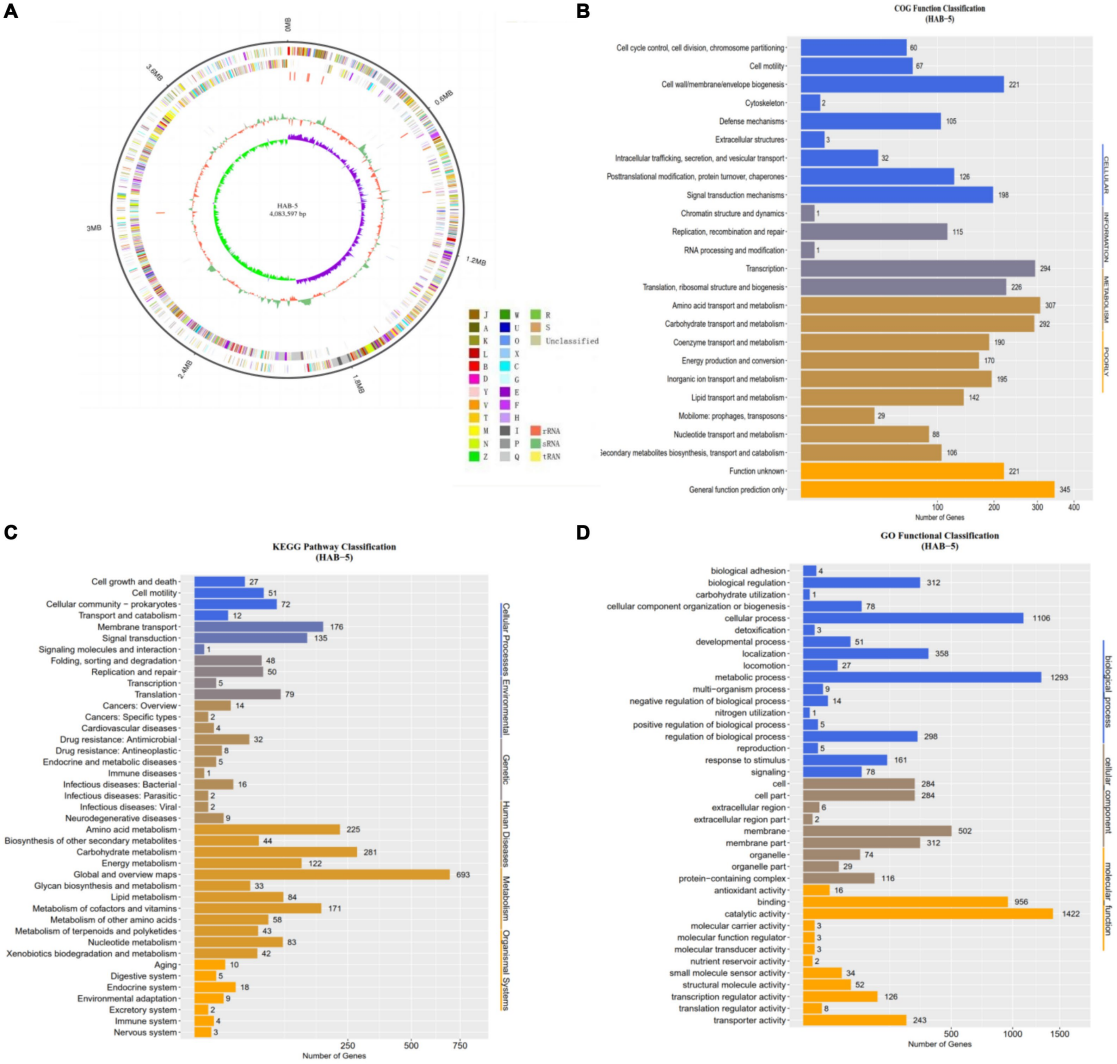
The complete genome sequence of *Bacillus atrophaeus* HAB-5 **(A)** is depicted with the following labeling from outside to inside: contigs, COGs on the forward strand; CDS, tRNAs, and rRNAs on the forward strand; CDS, tRNAs, and rRNAs on the reverse strand; COGs on the reverse strand; GC content; and GC skew. The Cluster of Orthologous Groups (COGs) analysis **(B)** of *B. atrophaeus* HAB-5 genes categorizes them into four groups: metabolism, cellular processes, information, and poorly characterized. The Kyoto Encyclopedia of Genes and Genomes (KEGG) analysis **(C)** classifies these genes into six categories: cellular processes, environmental information processing, genetic information processing, human diseases, metabolism, and organismal systems. The gene ontology (GO) analysis **(D)** groups the genes into three categories: biological processes, cellular components, and molecular functions.

In the present study, complete genome sequencing of HAB-5 was performed using third generation Pacific Biosciences (PacBio) single-molecule real-time (SMRT) sequencing platform technology. A total of 1,314 MB of raw data were collected, and 1,182 MB of data were assembled. The whole genome was distributed on a 4,083,597-bp circular chromosome with an average GC content of 44.2%. The strain contains 599 protein-coding gene CDSs, including 59 telomer restriction fragment, 38 minisatellite DNA, 02 microsatellite DNA, 82 tRNA, 08 rRNA, 29sRNA, 01 prophage, and 01 CRISPR domain. The distribution of genes in the COG functional categories is presented in Figure 1A, and additional information about the genome statistics is presented in Table 1.

**Table 1:** The general genome features of *Bacillus atrophaeus* HAB-5.

Features	Value
Genome size (bp)	4,083,597
GC content (%)	44.21
Gen length (bp)	3,555,024
Genome Length (%)	87.06
Total number of genes	4,226
Total number of rRNA	08
Total number of tRNA	82
Total number of ncRNA	135
Total number of sRNA	29
Total number of minisatellite DNA	38
Total number of microsatellite DNA	02
CRISPR number	01
Prophage number	01

### Analysis of gene function annotations in *Bacillus atrophaeus* HAB-5

To examine the functional composition and diversity of proteins and accessory genes in HAB-5, we evaluated the Cluster of Orthologous Groups (COGS), Kyoto Encyclopedia of Genes and Genomes (KEGG), and Gene Ontology (GO). The COG database predicted that 3,005 (71.1%) genes were distributed into approximately 814 genes assigned to the cellular domain, roughly 637 genes were annotated for information, roughly 1,519 genes were assigned to metabolism, and roughly 566 genes were allocated for low quality (Figure 1B). A total of 2,504 (59.25%) genes were annotated using the KEGG database (Figure 1C). KEGG metabolic pathway annotation showed that ~693 genes encoding protein genes participate in metabolism, including amino acid metabolism (~225 genes), other secondary metabolites (~44 genes), carbohydrate metabolism (~281 genes), energy metabolism (~122 genes), glycan biosynthesis and metabolism (~33 genes), lipid metabolism (~84 genes), metabolism cofactor and vitamins (~171 genes), metabolism and other amino acids(~58 genes), metabolism of terpenoids and polyketides (~43 genes), nucleotide metabolism (~83 genes), and xenobiotic biodegradation and metabolism (~42 genes). In the GO analysis, a total of 2,436 (57.64%) protein-coding genes were annotated. The annotated genes were mainly distributed into three major categories: molecular function (MF; ~3,804 genes), biological process (BP; ~3,804 genes), and cellular component (CC; ~1,609 genes; Figure 1D).

### Comparative genome analysis

#### Phylogenetics of *Bacillus atrophaeus* HAB-5

**Figure 2:**
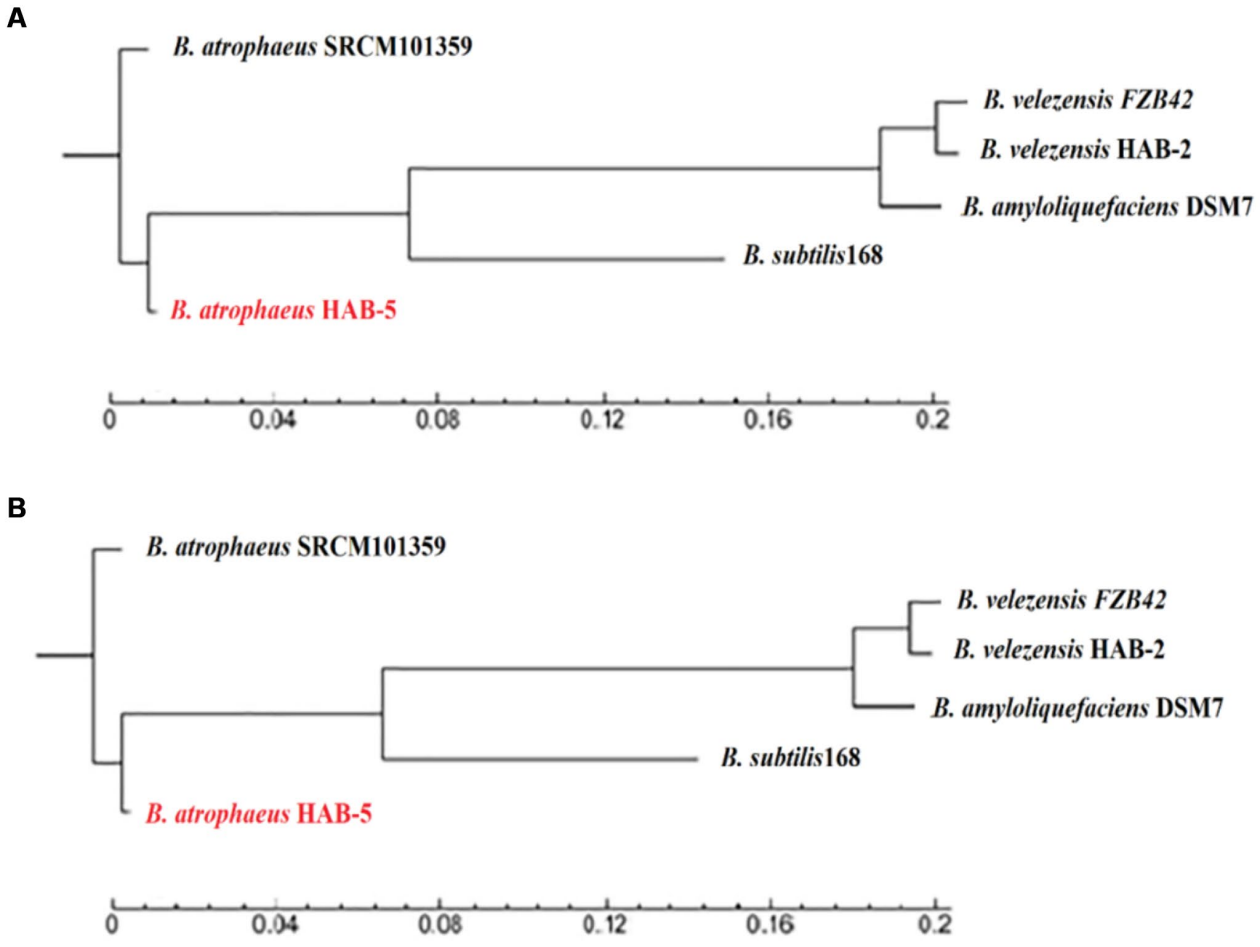
Examination of the phylogenetic tree for the *Bacillus* strains with *Bacillus atrophaeus* HAB-5. **(A)** The phylogenetic tree was built based on 16 s RNA of *Bacillus* strains and **(B)** the WGS phylogenetic tree of *B. atrophaeus* HAB-5, *B. atrophaeus* SRCM101359, *B. velezensis* FZB42, *B. velezensis* HAB-2, *B. amyloliquefaciens* DSM7, and *B. subtilis*168.

To determine the level of differences between strains HAB-5, DSM7, SRCM101359, 168, FZb42, and HAB-2, the 16S rRNA sequence of strain HAB-5 and other correlated sequences were obtained from NCBI for the construction of the phylogenetic association tree using MEGA 5.0. The phylogenetic relationship of HAB-5 was similar to that of SRCM101359 (Figure 2A). Additionally, WG-based phylogeny was constructed, which showed that the HAB-5 genome is closely linked to SRCM101359. However, lower phylogenetic relationships were found among the distinct branches from the other strains 168, DSM7, FZB42, and HAB-2 (Figure 2B).

#### Pan-genome identification and comparative analysis and gene families

**Figure 3:**
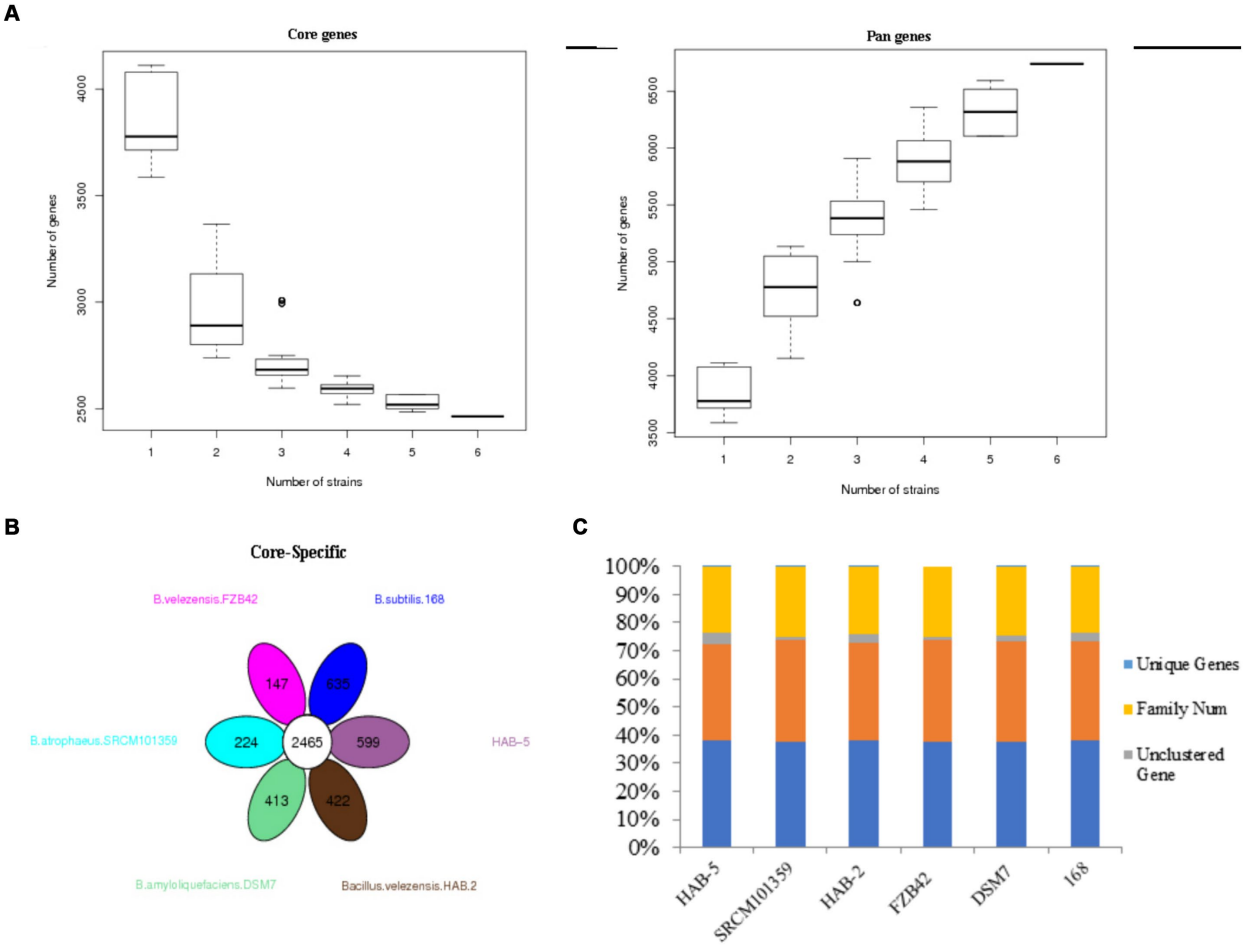
**(A)** Pan gene and core gene dilution curve, **(B)** conversed and specific gene counts (every ellipse shows strains, and numbers in the ellipse are specific genes; the white circle represents conserved genes among the six *Bacillus* strains), and **(C)** number of orthologs (unique genes, family number, unclustered genes, clustered genes, and gene number).

A bacterial pan-genome analysis was carried out. The pan-genome is made up of approximately 6,741 genes and shares approximately 2,465 genes as the core genome (Figure 3A), with HAB-5 having only 599 CDSs, followed by DSM7 (~413), SRCM101359 (~224), FZB42 (~147), HAB-2 (~422), and 168 (~635; Figure 3B). To compare the genes of the six strains, the genes family was used; strain FZb42 had the fewest genes (~3,687), strain 168 had the most genes (~4,233), and strain HAB-5 had the total genes (~4,226). As indicated by the table, the total number of unique genes was found in HAB-5 (~04) genes, which were followed by genes in DSM7 (~28), SRCM101359 (~03), FZb42 (~ 0), HAB-2 (~ 04), and 168 (~07; Figure 3C). These peculiar genes will aid in the identification of novel genes that may develop new roles.

#### Dispensable gene heat map

**Figure 4:**
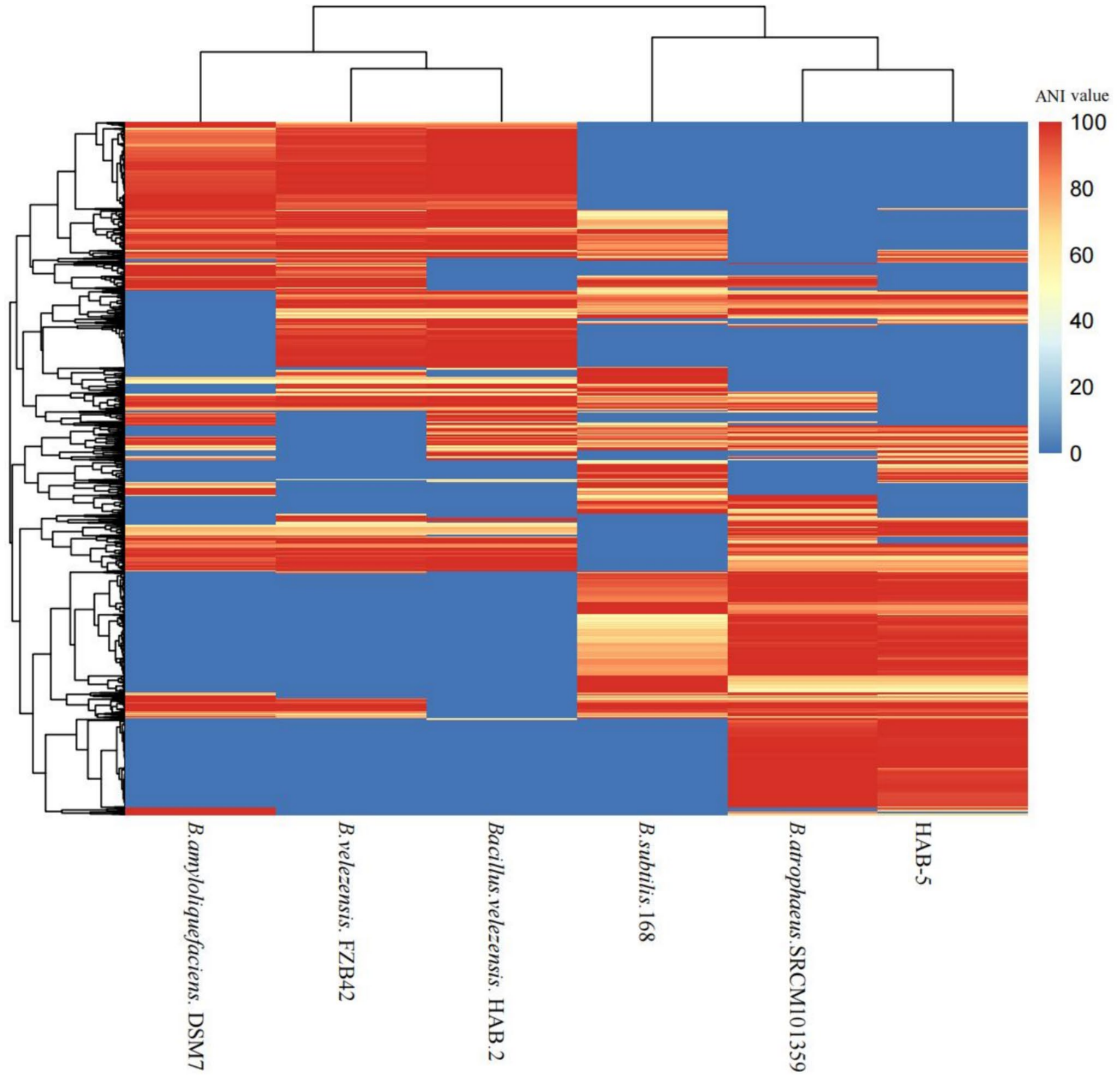
Dispensable gene heat map of the average nucleotide (ANI) value of the whole genome of the strain *Bacillus atrophaeus* HAB-5 and five other *Bacillus* strains.

The average nucleotide identity was examined using the dispensable gene heat map. The strain HAB-5 was most closely related to the strain SRCM101359. The average nucleotide identity of 168, DSM7, FZB42, and HAB-2 showed the least similarity with HAB-5. HAB-5 and SRCM101359 had the highest ANI identity (Figure 4). Together with a genome-to-genome distance calculator, average nucleotide identity has become a potent genome-based criterion for identifying species. It can reveal which genomes need to have their taxonomic and evolutionary positions altered or reclassified.

#### Structural distinction and collinearity analysis

We performed a collinearity analysis to compare the genomic similarities of HAB-5 with other strains (DSM7, SRCM101359, 168, FZb42, and HAB-2). The results showed that the HAB-5 genome demonstrated different synteny to 168 (Supplementary Figures S1A,B), followed by FZb42 (Supplementary Figures S1C,D), DSM7 (Supplementary Figures S1E,F), HAB-2 (Supplementary Figures S1G,H), and SRCM101359 (Supplementary Figures S1I,J). HAB-5 showed the highest levels of nucleotide and amino acid synteny with the SRCM101359 genome; representatives of their evolutionary stages were the closest, and their genomes were more related.

#### Genetic basis for producing the antimicrobial activity of *Bacillus atrophaeus* HAB-5

**Figure 5:**
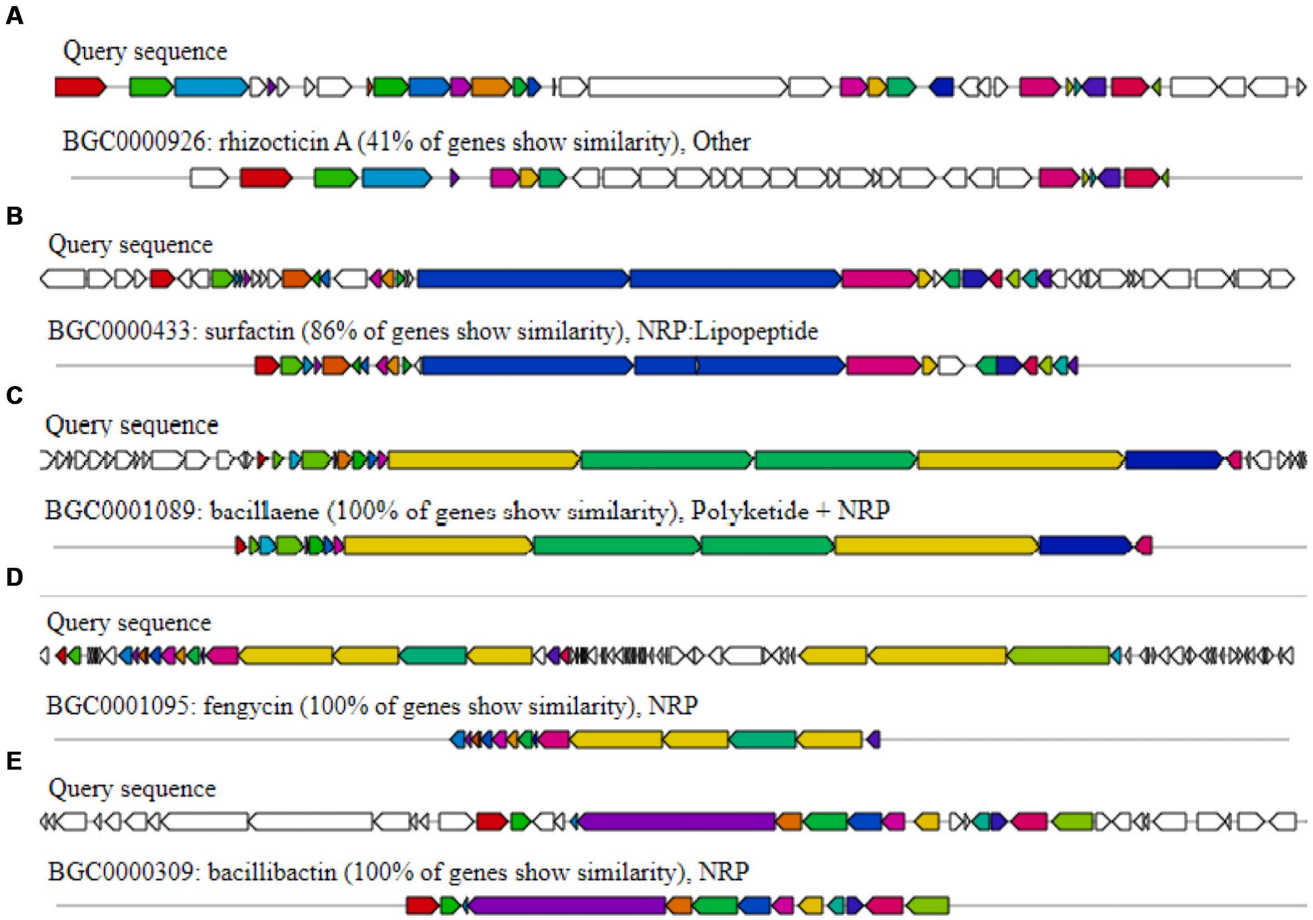
Secondary metabolite gene clusters with antimicrobial activity in *Bacillus atrophaeus* HAB-5 identified by antiSMASH **(A)** rhizocticin A, **(B)** surfactin, **(C)** bacillaene, **(D)** fengycin, and **(E)** bacillibactin.

It was determined that the HAB-5 strain had inhibitory effects on 22 plant pathogenic fungi, viruses, and activities that promoted plant growth, suggesting the presence of gene clusters that promote both plant growth and antimicrobial activity (Rajaofera et al., 2018, 2019, 2020). AntiSMASH predicted a total of 11 gene clusters in the HAB-5 genome (Blin et al., 2013). These include three gene clusters that encode non-ribosomal peptides (NRPs), two that encode Transat-pks, two that encode terpenes, one that encodes lanthipeptide, one that encodes type III polyketide synthase (T3PKS), one that encodes RiPP, and one that encodes thiopeptide. The gene clusters such as bacillaene, fengycin, and bacillibactin showed 100% amino acid sequence homology. While clusters 3, 4, 7, 8, 10, and 11 exhibited no similarity with known gene clusters, gene clusters 2 and 1 showed 86% amino acid similarity with surfactin synthetases and 41% similarity with rhizoctin biosynthetic genes in HAB-5. Identifications were made of the main biosynthetic genes, additional biosynthetic genes, transport-related genes, regulatory genes, and other genes (Figure 5; Table 2). Furthermore, the fengycin gene cluster includes the core genes involved in biosynthesis, such as *yngE, yngF, yngG, yngH, yngI, yngJ, yngK, yngL, dacC, fenA, fenB, fenC, fenD,* and *fenE*. Among the essential biosynthetic genes in the bacillaene gene cluster are *baeB, baeC, baeD, baeE, acpK, baeL, baeR, baeS, baeN, baeM, baeJ, baeI, baeG,* and *baeH*, and the bacillibactin gene cluster’s core biosynthesis genes include *besA, dhbA, dhbB, dhbC, dhbE, dhbF,* and *mbtH*. Finally, the core biosynthetic genes comprise the surfactin gene cluster (*aat, Ycxc, ycxD, sfp, yczE, yckI, yckJ, yciC, yx01, yckC, yckD, yckE, nin, hxlA, hxlB, hxlR, xy02, srfAA, srfAB, comS, srfAC,* and *srfAD*; Table 3).

**Table 2:** The potential secondary metabolites gene clusters in *B. atrophaeus* HAB-5.

Cluster	Types	From	To	Correlated gene clusters known	Similarities	MIBIG BGC-ID
1	NRPs	194,994	238, 980	Rhizoctin	41%	BGC0000926-c1
2	NRPs	372,059	435,835	Surfactin NRPs, a lipopeptide	86%	BGC0000433-c1
3	Lanthipeptide Class-iii	536,865	563,021	Unknown	-	-
4	Terpene	1,150,853	1,171,010	Unknown	-	-
5	TranAT-PKS	1,771,678	1,876,385	Bacillaene	100%	BGC0001089-c1
6	NRPs TranAT-PKS	1,967,734	2,113,080	Fengycin	100%	BGC0001095-c1
7	Terpene	2,131,456	2,153,345	-	-	-
8	T3PKS	2,226,585	2,267,688		-	-
9	NRPs	3,141,074	3,188,258	Bacillibactin	100%	BGC0001095-c1
10	Thiopeptide, a lap	3,206,638	3,236,740			
11	RiPP-like	3,959,475	3,971,658	-	-	-

**Table 3:** Genes and gene clusters of predicted active metabolites detected in the genome of *B. atrophaeus* HAB-5.

Metabolite	Gene and gene clusters	Function	Antimicrobial activity
Fengycin	yngE, yngF, yngG, yngH, yngI, yngJ, yngK, yngL, dacC, fenA, fenB, fenC, fenD, and fenE	Antibiotic Induction of ISR	Fungi
Bacillaene	baeB, baeC, baeD, baeE, acpK, baeL, baeR, baeS, baeN, baeM, baeJ, baeI, baeG, and baeH	Antibiotic Induction of ISR	Bacteria
Bacillibactin	besA, dhbA, dhbB, dhbC, dhbEdhbF, and mbtH	Siderophore during iron deficiency in soil	Bacteria
Surfactin	aat, Ycxc, ycxD, sfp, yczE, yckI, yckJ, yciC, yx01, yckC, yckD, yckE, nin, hxlAhxlB, hxlR, xy02, srfAA, srfAB, comSsrfAC, and srfAD	Antibiotic Induction of ISR	Fungi

#### Genetic basis for the plant growth-promoting activity of *Bacillus atrophaeus* HAB-5

Beneficial rhizobacteria influence plant growth by affecting nutrient uptake. Most of the genes associated with promoting plant growth were identified in HAB-5. IAA is a significant phytohormone that regulates plant cell growth and tissue differentiation; there are some nice genes related to IAA biosynthesis that have been identified in strain HAB-5 (Supplementary Table S1). Iron, sulfur, phosphorus, and nitrogen are necessary for the growth and development of plants. It was predicted that the gene clusters (*nif3*-like, *glt, gln, gln, nad, nirB, nir, nas, nar, nar,* and *nar*) would be involved in nitrogen metabolism and fixation (Supplementary Table S2). A number of other genes involved in iron transportation (*fbpA, fetB, feuC, feuB, feuA,* and *fecD*; Supplementary Table S3), phosphate solubilization (*ispH, pstA, pstC, pstS, pstB, gltP,* and *phoH*; Supplementary Table S4), and sulfur metabolisms (*cysC, sat, cysK, cysS,* and *sulP*; Supplementary Table S5) were found in HAB-5’s genome. In the present research, we have identified two more genes encoding acetoin (*acuB* and *acuC*) and three genes encoding enzymes of the biosynthetic pathway from acetion dehydrogenase, such as *acoA, acoB*, and *acoR* (Supplementary Table S6).

### Comparative genome analysis of *Bacillus atrophaeus* HAB-5

The comparative gene analysis focused on plant growth promotion revealed that most of the analyzed genes were common across strains HAB-5, SRCM101359, DSM7, FZB42, HAB-2, and 168, while unique genes such as *YwkB* and *FeuB* were absent in strains DSM7, FZB42, and HAB-2 (Supplementary Table S7). According to the comparative gene analysis of volatile chemicals, the genomes of HAB-5 and SRCM101359 both contained genes for the enzymes acetion dehyrogenase (*acoA, acoB*, and *acoR*) and acetion (*acuB* and *acuC*). Three-hydroxy-2-butanone, acetolactate decarboxylase (*alsD*), acetolactate synthase (*alsS*), a transcriptional regulator (*alsR*), and 2,3-butanediol dehydrogenase (*bdh*) genes were predicted in the FZB42 and HAB-2, followed by the genes associated with acetion and 2,3-butanediol synthesis like acetolactate synthase (*alsS*), acetolatate decarboxylase (*budA*), and acetion dehyrogenase (*acoA, acoB, acoC,* and *acoR*), were identified in *B. subtilis* 168. Genes for 2, 3-butanediol (*butA* and *butC*) and acetoin (*acuA* and *acuC*) were also predicted in DSM7 (Supplementary Table S8).

## Discussion

Genome sequences were performed to ascertain the molecular basis of the mechanisms underlying the promotion of plant growth and the biocontrol capabilities of *B. atrophaeus* HAB-5, and a comparative genome analysis was conducted with other *Bacillus* strains. The genomic analysis showed that 1,182 (Mb) of clean data were created and that the HAB-5 genome contained a circular chromosome with a size of 4,083,597 bp and a GC content of 43.36%. Additionally, the genome did not contain any plasmids. A comparative analysis showed that the genome size of HAB-5 (4,083,597 bp) was larger than that of DSM7 (3,980,199 bp), FZb42 (3,918,596 bp), and HAB-2 (3,894,648 bp), but it was still smaller than that of 168 (4,215,606 bp) and SRCM101359 (4,180,819 bp). The GC content of HAB-5 (44.21%) was higher than that of 168 (43.1%) and SRCM101359 (43%) but it was lower than that of FZb42 (46.6%), HAB-2 (46.6%), and DSM7 (46.1%). To determine the relationship between HAB-5 and other strains, a phylogenetic tree based on the 16SrRNA gene sequence and a phylogenomic tree were constructed. The results showed that HAB-5 is closely related to SRCM101359 and showed the highest similarity.

In previous studies, many plant growth-promoting bacteria were analyzed at the whole-genome level to gain an in-depth understanding of PGP mechanisms in bacteria such as *Pseudomonas aeruginosa* B18 (Singh et al., 2021), *B. velezensis* HAB-2 (Xu et al., 2020), *B. megaterium* BM89 and *B. subtilis* BS87 (Chandra et al., 2021), *K.* var*iicola* UC4115 (Guerrieri et al., 2021), and *Streptomyces* (Subramaniam et al., 2020). Numerous genes involved in biocontrol and growth-promoting activities in plants have been identified by whole genome sequencing. The presence of antimicrobial genes was also revealed by an analysis of the *B. atrophaeus* genome for the presence of secondary metabolites. *Bacillus atrophaeus* strains are remarkably capable of producing secondary metabolites with antimicrobial compounds, such as terpenes, polyketides, and non-ribosomally synthesized peptides (NRPs; Liu et al., 2012; Chan et al., 2013; Ma et al., 2018). In a previous study, genomic research revealed that *B. atrophaeus* L193 carries a cluster of genes known as non-ribosomal peptide synthetases. These genes include *fenC, srfA-A, BmyB,* and *ituD*, which are involved in the production of surfactin, fengycin, bacillomycin, and iturin (Rodríguez et al., 2018). Eight gene clusters that produce antimicrobial secondary metabolites, such as surfactin, bacillaene, fengycin, and bacillibactin, were found in the genome study of *B. atrophaeus* GQJK17 (Ma et al., 2018).

In the present studies, the whole genome sequencing of HAB-5 identified the genes encoding for novel antimicrobial peptides associated with its biocontrol properties. There were 11 gene clusters predicted in the genome of HAB-5: three gene clusters encoding for NRPS (non-ribosomal peptide synthetases), two gene clusters encoding for Transat-pks, two gene clusters encoding for terpene, one encoding for lanthipeptide, one gene cluster for T3PKS, one encoding RiPP, and one encoding thiopeptide that synthesized bacillaene, fengycin, bacillibactin, surfactin, and rhizocticin A. These secondary metabolites show antifungal and antibacterial activities against plant pathogens. According to Ongena and Jacques (2008), surfactin possesses antibacterial and antifungal properties, and fengycin and rhizocticin exhibit antifungal properties (Kugler et al., 1990). Bacillaene exhibits antimicrobial activity against many types of plant-harmful bacteria and fungi (Patel et al., 1995; Um et al., 2013; Müller et al., 2014), and two of the HAB-5 secondary metabolites gene clusters that may produce terpenes were identified; however, the other four are yet unknown. Terpenes are large, diversified, naturally occurring organic compounds that are present in bacteria, fungi, plants, and animals. They have a variety of medicinal uses and can be added to food and cosmetic products. They also have antifungal and anticarcinogenic characteristics (Zhao et al., 2016). Additionally, it plays a significant role in defending numerous plant, animal, and microbe species from infections and insects, as well as transmitting messages to non-specific and mutuality regarding the existence of food, partners, and adversaries, as well as from abiotic and biotic stressors (Gershenzon and Dudareva, 2007). Furthermore, the HAB-5 genome showed an amazing capacity to create bacillibactin, a type of siderophore that is characterized by short peptide molecules with functional groups and a side chain that can provide a set of ligands to coordinate ferric ions (Crosa and Walsh, 2002). Bacillibactin is a kind of strong siderophore that increases the absorption of ferric ions in soil for plant growth and to secrete volatile compounds (Nithyapriya et al., 2021). Furthermore, the gene cluster of Bacillibactin strain HAB-5 also contains other genes that promote plant growth and codify useful substances such as butanone, protease, phytase, and phosphatase (Ma et al., 2018; Rajaofera et al., 2020). Among the metabolites, VOCs have gained great attention for their potential in the control of plant pathogens. It has been reported that the strain HAB-5 produces a variety of VOCs, which have strong antifungal effects, inhibiting the growth of *C. gloeosporioides* (Rajaofera et al., 2019). Besides, HAB-5 has detected volatile chemical-producing genes such as *acoA, acoB, acoR, acuB,* and *acuC*. Acetion, one of the active bacterial volatile compounds, was released to stimulate the induced systemic resistance (ISR) of plants (Zhang et al., 2015).

Whole genome sequencing has revealed several genes linked to the promotion of plant growth, including chitinase, nitrogen fixation, phosphorus solubilization, auxin synthesis, iron acquisition, potassium, and IAA (Subramaniam et al., 2020; Chandra et al., 2021; Guerrieri et al., 2021; Iqbal et al., 2021; Singh et al., 2021). Most of the genes linked to promoting plant growth were found in HAB-5 after the genome HAB-5 annotation was completed in the current study. IAA is an important phytohormone that controls cell enlargement and tissue differentiation in plants. Genes, such as *trpA, trpB, trpC, trpD, trpE, trpS, ywkB, miaA,* and *nadE,* that contribute to the production of IAA, ethylene, and ammonia have been predicted in strain HAB-5. The occurrence of the gene clusters (*trpABCDEG, trpBCDES, trpABCDR, trpABD*, and *trpBE*), which are responsible for IAA production, also supports our findings from whole genome analysis of strains *P. aeruginosa* B18 (Singh et al., 2021), Rhizobacteria (Gupta et al., 2014), *B. cereus* T4S (Babalola et al., 2021), *Sphingomona* ssp. LK11 (Asaf et al., 2018), and *Enterobacter roggenkampii* ED5 (Guo et al., 2020). Similarly, we identified the genes involved in nitrogen fixation (*nif3*-like, *gltP*), nitrogen metabolism (*gltX, glnR, glnA*), and nitrogen regulation dissimulator nitrate (*nadR, nirB, nirD, nasD, narl, narH, narJ,* and *nark*) in the genome of HAB-5. Bacteria contain several iron transporters, e.g., *Ybt, Feo, Efe, Yfe, Fet,* and *Fhu* in *Yersinia pestis* (Forman et al., 2007). Among such iron transporters, the Fhu system—a siderophore receptor called FhuD—participates in siderophore (hydroxamate)-dependent iron (III) transport and was initially discovered in *Escherichia coli* (Kammler et al., 1993). In the present studies, several other genes (*fbpA, fetB, feuC, feuB, feuA*, and *fecD*) involved in iron transportation were also identified in HAB-5, according to the current research. Additionally, comparable findings were noted for the following bacteria: *E. coli* (*FeoAB*; Kammler et al., 1993), *Shigella fexneri* (*FecIRABCDE*; Luck et al., 2001), *Staphylococcus* (*SirABC*; Dale et al., 2004), and *Yersinia pestis* (*Efe, Yfe*, and *Fet*; Forman et al., 2007). Additionally, we discovered a few genes related to phosphate uptake and solubilization, some of which have been thoroughly investigated. These genes include *pst* (specific transporter) and *pho* (alkaline phosphate; Gebhard et al., 2006; Martín and Liras, 2021). The HAB-5 genome contained the phosphate solubilization-related genes (*ispH, pstA, pstC, pstS, pstB, gltP*, and *phoH*). Moreover, the genomes of *Streptomyces* and *Mycobacterium tuberculosis* have been revealed to contain *pstA, pstC, pstS,* and *pstB*, as well as *phoA, phoC,* and *phoX*, respectively (Gebhard et al., 2006; Martín and Liras, 2021). Furthermore, the HAB-5 genome contained genes associated with sulfur metabolisms (*cysC, sat, cysK, cysS*, and *sulP*), which have been found and described as sulfur metabolisms in *Bacillus*.

## Conclusion

Our study findings show that whole genome sequencing of *B. atrophaeus* HAB-5 generated 4,083,597 bp. A comparative genomic analysis of the HAB-5 strain with other *Bacillus* strains revealed its genome similarity to SRCM101359. Through genome mining, HAB-5 was found to harbor several antimicrobial secondary metabolites contributing to its biocontrol activities and demonstrated multiple genes related to IAA phytohormones, iron, sulfur, phosphate solubilization, and nitrogen fixation. These results will contribute to in-depth research on plant growth promotion and biocontrol mechanisms.

## Data availability statement

The datasets presented in this study can be found in online repositories. The names of the repository/repositories and accession number(s) can be found in the article/Supplementary material.

## Author contributions

GD: Data curation, Investigation, Methodology, Writing – original draft, Writing – review & editing. HW: Resources, Writing – review & editing, Methodology, Software. FR: Resources, Writing – review & editing. PJ: Resources, Writing – review & editing. PX: Methodology, Resources, Writing – review & editing, Validation. MA: Resources, Writing – review & editing. AQ: Resources, Writing – review & editing, Data curation, Formal analysis. AK: Writing – review & editing, Methodology, Resources. WM: Conceptualization, Funding acquisition, Supervision, Writing – review & editing, Project administration.

## Footnotes

^1^ https://github.com/robotoD/GenoVi

^2^ http://www.cbcb.umd.edu/software/glimmer

^3^ https://discover.nci.nih.gov/cimminer

## References

[ref1] Asaf S. Khan A. L. Khan M. A. Al-Harrasi A. Lee I. J. (2018). Complete genome sequencing and analysis of endophytic *Sphingomonas* sp. LK11 and its potential in plant growth. 3 Biotech 8:389. doi: 10.1007/s13205-018-1403-z, PMID: 30175026 PMC6111035

[ref2] Babalola O. O. Adeleke B. S. Ayangbenro A. S. (2021). Whole genome sequencing of sunflower root-associated *Bacillus cereus*. Evol. Bioinformatics Online 17:11769343211038948. doi: 10.1177/11769343211038948, PMID: 34421294 PMC8375328

[ref3] Bais H. P. Fall R. Vivanco J. M. (2004). Biocontrol of *Bacillus subtilis* against infection of Arabidopsis roots by *Pseudomonas syringae* is facilitated by biofilm formation and surfactin production. Plant Physiol. 134, 307–319. doi: 10.1104/pp.103.028712, PMID: 14684838 PMC316310

[ref4] Bard J. Winter R. (2000). Gene ontology: a tool for the unification of biology. Nat. Genet. 25, 25–29. doi: 10.1038/75556 10802651 PMC3037419

[ref5] Blin K. Medema M. H. Kazempour D. Fischbach M. A. Breitling R. Takano E.. (2013). antiSMASH 2.0--a versatile platform for genome mining of secondary metabolite producers. Nucleic Acids Res. 41, W204–W212. doi: 10.1093/nar/gkt449, PMID: 23737449 PMC3692088

[ref6] Blin K. Shaw S. Augustijn H. E. Reitz Z. L. Biermann F. Alanjary M.. (2023). antiSMASH 7.0: new and improved predictions for detection, regulation, chemical structures and visualisation. Nucleic Acids Res. 51, W46–W50. doi: 10.1093/nar/gkad344, PMID: 37140036 PMC10320115

[ref7] Bornscheuer U. T. (2016). Microbiology. Feeding on plastic. Science 351, 1154–1155. doi: 10.1126/science.aaf2853 26965614

[ref8] Cabanettes F. Klopp C. (2018). D-Genies: dot plot large genomes in an interactive, efficient and simple way. PeerJ 6:e4958. doi: 10.7717/peerj.4958, PMID: 29888139 PMC5991294

[ref9] Chan W. Y. Dietel K. Lapa S. V. Avdeeva L. V. Borriss R. Reva O. N. (2013). Draft genome sequence of *Bacillus atrophaeus* UCMB-5137, a plant growth-promoting Rhizobacterium. Genome Announc. 1, e00233–e00213. doi: 10.1128/genomeA.00233-13, PMID: 23788535 PMC3707584

[ref10] Chandra A. Chandra P. Tripathi P. (2021). Whole genome sequence insight of two plant growth-promoting bacteria (*B. subtilis* BS87 and *B. megaterium* BM89) isolated and characterized from sugarcane rhizosphere depicting better crop yield potentiality. Microbiol. Res. 247:126733. doi: 10.1016/j.micres.2021.126733, PMID: 33676313

[ref11] Chandran H. Meena M. Sharma K. (2020). Microbial biodiversity and bioremediation assessment through omics approaches. Front. Environ. Chem. 1:570326. doi: 10.3389/fenvc.2020.570326

[ref12] Chin C. S. Alexander D. H. Marks P. Klammer A. A. Drake J. Heiner C.. (2013). Nonhybrid, finished microbial genome assemblies from long-read SMRT sequencing data. Nat. Methods 10, 563–569. doi: 10.1038/nmeth.2474, PMID: 23644548

[ref13] Chin C. S. Peluso P. Sedlazeck F. J. Nattestad M. Concepcion G. T. Clum A.. (2016). Phased diploid genome assembly with single-molecule real-time sequencing. Nat. Methods 13, 1050–1054. doi: 10.1038/nmeth.4035, PMID: 27749838 PMC5503144

[ref14] Chowdhury S. P. Hartmann A. Gao X. Borriss R. (2015). Biocontrol mechanism by root-associated *Bacillus amyloliquefaciens* FZB42—a review. Front. Microbiol. 6:780. doi: 10.3389/fmicb.2015.00780, PMID: 26284057 PMC4517070

[ref15] Chun B. H. Kim K. H. Jeon H. H. Lee S. H. Jeon C. O. (2017). Pan-genomic and transcriptomic analyses of *Leuconosto cmesenteroides* provide insights into its genomic and metabolic features and roles in kimchi fermentation. Sci. Rep. 7:11504. doi: 10.1038/s41598-017-12016-z, PMID: 28912444 PMC5599536

[ref16] Chun B. H. Kim K. H. Jeong S. E. Jeon C. O. (2019). Genomic and metabolic features of the *Bacillus amyloliquefaciens* group-*B. amyloliquefaciens, B. Velezensis, and B. siamensis-*revealed by pan-genome analysis. Food Microbiol. 77, 146–157. doi: 10.1016/j.fm.2018.09.001, PMID: 30297045

[ref17] Crosa J. H. Walsh C. T. (2002). Genetics and assembly line enzymology of siderophore biosynthesis in bacteria. Microbiol. Mol. Biol. Rev. 66, 223–249. doi: 10.1128/MMBR.66.2.223-249.2002, PMID: 12040125 PMC120789

[ref18] Dale S. E. Sebulsky M. T. Heinrichs D. E. (2004). Involvement of SirABC in iron-siderophore import in *Staphylococcus aureus*. J. Bacteriol. 186, 8356–8362. doi: 10.1128/JB.186.24.8356-8362.2004, PMID: 15576785 PMC532444

[ref19] Darling A. C. Mau B. Blattner F. R. Perna N. T. (2004). Mauve: multiple alignment of conserved genomic sequence with rearrangements. Genome Res. 14, 1394–1403. doi: 10.1101/gr.2289704, PMID: 15231754 PMC442156

[ref20] Delcher A. L. Kasif S. Fleischmann R. D. Peterson J. White O. Alzberg S. L. (1999). Alignment of whole genomes. Nucleic Acids Res. 27, 2369–2376. doi: 10.1093/nar/27.11.2369, PMID: 10325427 PMC148804

[ref21] Edgar R. C. (2004). MUSCLE: multiple sequence alignment with high accuracy and high throughput. Nucleic Acids Res. 32, 1792–1797. doi: 10.1093/nar/gkh340, PMID: 15034147 PMC390337

[ref22] Fan B. Wang C. Song X. Ding X. Wu L. Wu H.. (2018). *Bacillus velezensis* FZB42 in 2018: the gram-positive model strain for plant growth promotion and biocontrol. Front. Microbiol. 9:2491. doi: 10.3389/fmicb.2018.02491, PMID: 30386322 PMC6198173

[ref23] Forman S. Nagiec M. J. Abney J. Perry R. D. Fetherston J. D. (2007). Analysis of the aerobactin and ferric hydroxamate uptake systems of *Yersinia pest* is. Microbiology 153, 2332–2341. doi: 10.1099/mic.0.2006/004275-0, PMID: 17600077

[ref24] García-Fraile P. Menéndez E. Rivas R. (2015). Role of bacterial biofertilizers in agriculture and forestry. AIMS Bioengineer 2, 183–205. doi: 10.3934/bioeng.2015.3.183

[ref25] Gebhard S. Tran S. L. Cook G. M. (2006). The Phn system of *Mycobacterium smegmatis*: a second high-affinity ABC-transporter for phosphate. Microbiology 152, 3453–3465. doi: 10.1099/mic.0.29201-0, PMID: 17074913

[ref26] Gershenzon J. Dudareva N. (2007). The function of terpene natural products in the natural world. Nat. Chem. Biol. 3, 408–414. doi: 10.1038/nchembio.2007.5 17576428

[ref27] Guerrieri M. C. Fiorini A. Fanfoni E. Tabaglio V. Cocconcelli P. S. Trevisan M.. (2021). Integrated genomic and greenhouse assessment of a novel plant growth-promoting Rhizobacterium for tomato plant. Front. Plant Sci. 12:660620. doi: 10.3389/fpls.2021.660620, PMID: 33859664 PMC8042378

[ref28] Guo D. J. Singh R. K. Singh P. Li D. P. Sharma A. Xing Y. X.. (2020). Complete genome sequence of *Enterobacter roggenkampii* ED5, a nitrogen fixing plant growth promoting endophytic bacterium with biocontrol and stress tolerance properties, isolated from sugarcane root. Front. Microbiol. 11:580081. doi: 10.3389/fmicb.2020.580081, PMID: 33072048 PMC7536287

[ref29] Gupta A. Gopal M. Thomas G. V. Manikandan V. Gajewski J. Thomas G.. (2014). Whole genome sequencing and analysis of plant growth promoting bacteria isolated from the rhizosphere of plantation crops coconut, cocoa and arecanut. PLoS One 9:e104259. doi: 10.1371/journal.pone.0104259, PMID: 25162593 PMC4146471

[ref30] Guy L. Kultima J. R. Andersson S. G. (2010). genoPlotR: comparative gene and genome visualization in R. Bioinformatics 26, 2334–2335. doi: 10.1093/bioinformatics/btq413, PMID: 20624783 PMC2935412

[ref31] Huang H. Wu Z. Tian C. Liang Y. You C. Chen L. (2015). Identification and characterization of the endophytic bacterium *Bacillus atrophaeus* XW2, antagonistic towards *Colletotrichum gloeosporioides*. Ann. Microbiol. 65, 1361–1371. doi: 10.1007/s13213-014-0974-0

[ref32] Iqbal S. Vollmers J. Janjua H. A. (2021). Genome mining and comparative genome analysis revealed niche-specific genome expansion in antibacterial *Bacillus pumilus* strain SF-4. Genes 12:1060. doi: 10.3390/genes12071060, PMID: 34356076 PMC8303946

[ref33] Kammler M. Schön C. Hantke K. (1993). Characterization of the ferrous iron uptake system of *Escherichia coli*. J. Bacteriol. 175, 6212–6219. doi: 10.1128/jb.175.19.6212-6219.1993, PMID: 8407793 PMC206716

[ref34] Kanehisa M. Goto S. Kawashima S. Okuno Y. Hattori M. (2004). The KEGG resource for deciphering the genome. Nucleic Acids Res. 32, 277D–2280D. doi: 10.1093/nar/gkh063, PMID: 14681412 PMC308797

[ref35] Krzywinski M. Schein J. Birol I. Connors J. Gascoyne R. Horsman D.. (2009). Circos: an information aesthetic for comparative genomics. Genome Res. 19, 1639–1645. doi: 10.1101/gr.092759.109, PMID: 19541911 PMC2752132

[ref36] Kugler M. Loeffler W. Rapp C. Kern A. Jung G. (1990). Rhizocticin a, an antifungal phosphono-oligopeptide of *Bacillus subtilis* ATCC 6633: biological properties. Arch. Microbiol. 153, 276–281. doi: 10.1007/BF00249082, PMID: 2110446

[ref37] Li L. Stoeckert C. J. Roos D. S. (2003). OrthoMCL: identification of ortholog groups for eukaryotic genomes. Genome Res. 13, 2178–2189. doi: 10.1101/gr.1224503, PMID: 12952885 PMC403725

[ref38] Liu F. Sun W. Su F. Zhou K. Li Z. (2012). Draft genome sequence of the sponge-associated strain *Bacillus atrophaeus* C89, a potential producer of marine drugs. J. Bacteriol. 194:4454. doi: 10.1128/JB.00835-12, PMID: 22843588 PMC3416270

[ref39] Lowe T. M. Eddy S. R. (1997). tRNAscan-SE: a program for improved detection of transfer RNA genes in genomic sequence. Nucleic Acids Res. 25, 955–964. doi: 10.1093/nar/25.5.955, PMID: 9023104 PMC146525

[ref40] Luck S. N. Turner S. A. Rajakumar K. Sakellaris H. Adler B. (2001). Ferric dicitrate transport system (Fec) of *Shigella flexneri* 2a YSH6000 is encoded on a novel pathogenicity island carrying multiple antibiotic resistance genes. Infect. Immun. 69, 6012–6021. doi: 10.1128/IAI.69.10.6012-6021.2001, PMID: 11553538 PMC98729

[ref41] Ma J. Wang C. Wang H. Liu K. Zhang T. Yao L.. (2018). Analysis of the complete genome sequence of *Bacillus atrophaeus* GQJK17 reveals its biocontrol characteristics as a plant growth-promoting Rhizobacterium. Biomed. Res. Int. 2018:3542. doi: 10.1155/2018/9473542, PMID: 30046614 PMC6038694

[ref42] Maqbool O. Babri H. (2007). Hierarchical clustering for software architecture recovery. IEEE Trans. Softw. Eng. 33, 759–780. doi: 10.1109/TSE.2007.70732

[ref43] Martín J. F. Liras P. (2021). Molecular mechanisms of phosphate sensing, transport and Signalling in Streptomyces and related Actinobacteria. Int. J. Mol. Sci. 22:1129. doi: 10.3390/ijms22031129, PMID: 33498785 PMC7866108

[ref44] Medema M. H. Blin K. Cimermancic P. de Jager V. Zakrzewski P. Fischbach M. A.. (2011). antiSMASH: rapid identification, annotation and analysis of secondary metabolite biosynthesis gene clusters in bacterial and fungal genome sequences. Nucleic Acids Res. 39, W339–W346. doi: 10.1093/nar/gkr466, PMID: 21672958 PMC3125804

[ref45] Meena M. Swapnil P. Divyanshu K. Kumar S. Harish Tripathi Y. N. Zehra A.. (2020). PGPR-mediated induction of systemic resistance and physiochemical alterations in plants against the pathogens: current perspectives. J. Basic Microbiol. 60, 828–861. doi: 10.1002/jobm.202000370, PMID: 32815221

[ref46] Minkin I. Pham H. Starostina E. Vyahhi N. Pham S. (2013). C-Sibelia: an easy-to-use and highly accurate tool for bacterial genome comparison. F1000Res 2:258. doi: 10.12688/f1000research.2-258.v1, PMID: 25110578 PMC4111117

[ref47] Müller S. Strack S. N. Hoefler B. C. Straight P. D. Kearns D. B. Kirby J. R. (2014). Bacillaene and sporulation protect *Bacillus subtilis* from predation by *Myxococcus xanthus*. Appl. Environ. Microbiol. 80, 5603–5610. doi: 10.1128/AEM.01621-14, PMID: 25002419 PMC4178607

[ref48] Nandi T. Ong C. Singh A. P. Boddey J. Atkins T. Sarkar-Tyson M.. (2010). A genomic survey of positive selection in *Burkholderia pseudomallei* provides insights into the evolution of accidental virulence. PLoS Pathog. 6:e1000845. doi: 10.1371/journal.ppat.1000845, PMID: 20368977 PMC2848565

[ref49] Nithyapriya S. Sundaram Lalitha R. Z. Sayyed M. S. Reddy D. J. Dailin H. A. Enshasy E.. (2021). Production, purification, and characterization of Bacillibactin Siderophore of *Bacillus subtilis* and its application for improvement in plant growth and oil content in sesame. Sustain. For. 13:5394. doi: 10.3390/su13105394

[ref50] Ongena M. Jacques P. (2008). *Bacillus* lipopeptides: versatile weapons for plant disease biocontrol. Trends Microbiol. 16, 115–125. doi: 10.1016/j.tim.2007.12.009, PMID: 18289856

[ref51] Page A. J. Cummins C. A. Hunt M. Wong V. K. Reuter S. Holden M. T.. (2015). Roary: rapid large-scale prokaryote pan genome analysis. Bioinformatics 31, 3691–3693. doi: 10.1093/bioinformatics/btv421, PMID: 26198102 PMC4817141

[ref52] Patel P. S. Huang S. Fisher S. Pirnik D. Aklonis C. Dean L.. (1995). Bacillaene, a novel inhibitor of procaryotic protein synthesis produced by *Bacillus subtilis*: production, taxonomy, isolation, physico-chemical characterization and biological activity. J. Antibiot. 48, 997–1003. doi: 10.7164/antibiotics.48.997, PMID: 7592068

[ref53] Peng Y. Li S. J. Yan J. Tang Y. Cheng J. P. Gao A. J.. (2021). Research Progress on Phytopathogenic Fungi and their role as biocontrol agents. Front. Microbiol. 12:670135. doi: 10.3389/fmicb.2021.670135, PMID: 34122383 PMC8192705

[ref54] Rajaofera M. J. N. Jin P. F. Fan Y. M. Sun Q. Q. Huang W. K. Wang W. B.. (2018). Antifungal activity of the bioactive substance from *Bacillus atrophaeus* strain HAB-5 and its toxicity assessment on *Danio rerio*. Pestic. Biochem. Physiol. 147, 153–161. doi: 10.1016/j.pestbp.2017.06.006, PMID: 29933986

[ref55] Rajaofera M. J. N. Wang Y. Dahar G. Y. Jin P. Fan L. Xu L.. (2019). Volatile organic compounds of *Bacillus atrophaeus* HAB-5 inhibit the growth of *Colletotrichum gloeosporioides*. Pestic. Biochem. Physiol. 156, 170–176. doi: 10.1016/j.pestbp.2019.02.019, PMID: 31027577

[ref56] Rajaofera M. J. N. Wang Y. Jatoi Z. A. (2020). *Bacillus atrophaeus* HAB-5 secretion metabolites preventing occurrence of systemic diseases in tobacco plant. Eur. J. Plant Pathol. 156, 159–172. doi: 10.1007/s10658-019-01873-1

[ref57] Reva O. Chan W. Y. Lapa S. Borriss R. (2013). “Complete genome sequence of a plant growth promoting and crop protective strain of *Bacillus atrophaeus* UCMB-5137” in Endophytes for plant protection: The state of the art, *proceedings of the 5th international symposium on plant protection and plant health in Europe held at the Faculty of Agriculture and Horticulture (LGF)*. eds. Schneider C. Leifert C. Feldmann F. (Berlin-Dahlem, Germany: Humboldt University Berlin), 100.

[ref58] Richter M. Rosselló-Móra R. Oliver Glöckner F. Peplies J. (2016). JSpeciesWS: a web server for prokaryotic species circumscription based on pairwise genome comparison. Bioinformatics 32, 929–931. doi: 10.1093/bioinformatics/btv681, PMID: 26576653 PMC5939971

[ref59] Rodríguez M. Marín A. Torres M. Béjar V. Campos M. Sampedro I. (2018). Aphicidal activity of surfactants produced by *Bacillus atrophaeus* L193. Front. Microbiol. 9:3114. doi: 10.3389/fmicb.2018.03114, PMID: 30619189 PMC6305586

[ref60] Samaras A. Nikolaidis M. Antequera-Gómez M. L. Cámara-Almirón J. Romero D. Moschakis T.. (2021). Whole genome sequencing and root colonization studies reveal novel insights in the biocontrol potential and growth promotion by *Bacillus subtilis* MBI 600 on cucumber. Front. Microbiol. 11:600393. doi: 10.3389/fmicb.2020.600393, PMID: 33510723 PMC7837180

[ref61] Singh P. Singh R. K. Guo D. J. Sharma A. Singh R. N. Li D. P.. (2021). Whole genome analysis of sugarcane root-associated endophyte *Pseudomonas aeruginosa* B18-a plant growth-promoting bacterium with antagonistic potential against *Sporisorium scitamineum*. Front. Microbiol. 12:628376. doi: 10.3389/fmicb.2021.628376, PMID: 33613496 PMC7894208

[ref62] Subramaniam G. Thakur V. Saxena R. K. Vadlamudi S. Purohit S. Kumar V.. (2020). Complete genome sequence of sixteen plant growth promoting *Streptomyces* strains. Sci. Rep. 10:10294. doi: 10.1038/s41598-020-67153-9, PMID: 32581303 PMC7314817

[ref63] Syed Ab Rahman S. F. Singh E. Pieterse C. M. J. Schenk P. M. (2018). Emerging microbial biocontrol strategies for plant pathogens. Plant Sci. 267, 102–111. doi: 10.1016/j.plantsci.2017.11.012 29362088

[ref64] Tamura K. Peterson D. Peterson N. Stecher G. Nei M. Kumar S. (2011). MEGA5: molecular evolutionary genetics analysis using maximum likelihood, evolutionary distance, and maximum parsimony methods. Mol. Biol. Evol. 28, 2731–2739. doi: 10.1093/molbev/msr121, PMID: 21546353 PMC3203626

[ref65] Tatusov R. L. Fedorova N. D. Jackson J. D. Jacobs A. R. Kiryutin B. Koonin E. V.. (2003). The COG database: an updated version includes eukaryotes. BMC Bioinformatics 4:41. doi: 10.1186/1471-2105-4-41, PMID: 12969510 PMC222959

[ref66] Tudi M. Daniel Ruan H. Wang L. Lyu J. Sadler R. Connell D.. (2021). Agriculture development, pesticide application and its impact on the environment. Int. J. Environ. Res. Public Health 18:1112. doi: 10.3390/ijerph18031112, PMID: 33513796 PMC7908628

[ref67] Um S. Fraimout A. Sapountzis P. Oh D. C. Poulsen M. (2013). The fungus-growing termite *Macrotermes natalensis* harbors bacillaene-producing *Bacillus* sp. that inhibit potentially antagonistic fungi. Sci. Rep. 3:3250. doi: 10.1038/srep03250, PMID: 24248063 PMC3832938

[ref68] Vanittanakom N. Loeffler W. Koch U. Jung G. (1986). Fengycin--a novel antifungal lipopeptide antibiotic produced by *Bacillus subtilis* F-29-3. J. Antibiot. 39, 888–901. doi: 10.7164/antibiotics.39.888 3093430

[ref69] Walker B. J. Abeel T. Shea T. Priest M. Abouelliel A. Sakthikumar S.. (2014). Pilon: an integrated tool for comprehensive microbial variant detection and genome assembly improvement. PLoS One 9:e112963. doi: 10.1371/journal.pone.0112963, PMID: 25409509 PMC4237348

[ref70] Wang Y. Sun Z. Zhao Q. Yang X. Li Y. Zhou H.. (2024). Whole-genome analysis revealed the growth-promoting and biological control mechanism of the endophytic bacterial strain *Bacillus halotolerans* Q2H2, with strong antagonistic activity in potato plants. Front. Microbiol. 14:1287921. doi: 10.3389/fmicb.2023.1287921, PMID: 38235428 PMC10792059

[ref71] Wang K. Zhao Y. Wang X. Qu C. Miao J. (2020). Complete genome sequence of *Bacillus* sp. N1-1, a κ-selenocarrageenan degrading bacterium isolated from the cold seep in the South China Sea. Mar. Genomics 54:100771. doi: 10.1016/j.margen.2020.100771, PMID: 32273179

[ref72] Weber T. Blin K. Duddela S. Krug D. Kim H. U. Bruccoleri R.. (2015). antiSMASH 3.0-a comprehensive resource for the genome mining of biosynthetic gene clusters. Nucleic Acids Res. 43, W237–W243. doi: 10.1093/nar/gkv437, PMID: 25948579 PMC4489286

[ref73] Woo S. M. Kim S. D. (2008). Structural identification of siderophore AH18 from *Bacillus subtilis* AH18, a biocontrol agent of *Phytophthora* blight disease in red-pepper. Korean J. Microbiol. Biotechnol. 36, 326–335. doi: 10.1007/s11259-010-9358-5

[ref74] Xiong H. Li Y. Cai Y. Cao Y. Wang Y. (2015). Isolation of *Bacillus amyloliquefaciens* JK6 and identification of its lipopeptides surfactin for suppressing tomato bacterial wilt. RSC Adv. 5, 82042–82049. doi: 10.1039/C5RA13142A

[ref75] Xu P. Xie S. Liu W. Jin P. Wei D. Yaseen D. G.. (2020). Comparative genomics analysis provides new strategies for bacteriostatic ability of *Bacillus velezensis* HAB-2. Front. Microbiol. 11:594079. doi: 10.3389/fmicb.2020.594079, PMID: 33281792 PMC7705179

[ref76] Yu X. Ai C. Xin L. Zhou G. (2011). The siderophore-producing bacterium, *Bacillus subtilis* CAS15, has a biocontrol effect on *fusarium* wilt and promotes the growth of pepper. Eur. J. Soil Biol. 47, 138–145. doi: 10.1016/j.ejsobi.2010.11.001

[ref77] Zhang X. Li B. Wang Y. Guo Q. Lu X. Li S.. (2013). Lipopeptides, a novel protein, and volatile compounds contribute to the antifungal activity of the biocontrol agent *Bacillus atrophaeus* CAB-1. Appl. Microbiol. Biotechnol. 97, 9525–9534. doi: 10.1007/s00253-013-5198-x, PMID: 24013222

[ref78] Zhang N. Yang D. Wang D. Miao Y. Shao J. Zhou X.. (2015). Whole transcriptomic analysis of the plant-beneficial rhizobacterium *Bacillus amyloliquefaciens* SQR9 during enhanced biofilm formation regulated by maize root exudates. BMC Genomics 16:685. doi: 10.1186/s12864-015-1825-5, PMID: 26346121 PMC4562157

[ref79] Zhao J. Bao X. Li C. Shen Y. Hou J. (2016). Improving mono terpene geraniol production through geranyl diphosphate synthesis regulation in *Saccharomyces cerevisiae*. Appl. Microbiol. Biotechnol. 100, 4561–4571. doi: 10.1007/s00253-016-7375-1, PMID: 26883346

[ref80] Zhao Y. Wu J. Yang J. Sun S. Xiao J. Yu J. (2012). PGAP: pan-genomes analysis pipeline. Bioinformatics 28, 416–418. doi: 10.1093/bioinformatics/btr655, PMID: 22130594 PMC3268234

