# Supplementary material for: Comparative genomic analysis of Bacillus atrophaeus HAB-5 reveals genes associated with antimicrobial and plant growth-promoting activities
Source: Front Microbiol. 2024 Jun 26;15:1384691. doi: 10.3389/fmicb.2024.1384691 (PMC11233526; doi:10.3389/fmicb.2024.1384691)
Supplement: Supplementary file 1 [file Table_1.DOCX]

**Supplementary Table.05. Genes involved in modulation of plant hormones**

**Pathway Genes Protein coded by the genes Identity**

IAA production trpA, Tryptophan synthase subunit alpha trpA 98.13%

L-tryptophan trpB, Tryptophan synthase subunit alpha trpB 98.50%

trpC, Indole-3-glycerol phosphate synthase trpC 98.67%

trpD, Anthranilate phosphoribosyltransferase 97.94%

trpE, Anthranilate synthase component I 98.13%

trpS Tryptophan-tRNA ligase 98.69%

IPA pathway ywkB Auxin efflux carrier 83.21%

Cytokinin

biosynthesis and miaA Adenosinde-37-n6-dimethylallyltransferase 96.83%

transformation

Ammonia production nadE Ammonia-dependent NAD (+) synthase 98.17%

**Supplementary Table.06. Genes involved in nitrogen fixing and nitrogen metabolism**

**Pathway Genes Product Identity**

Nitrogen fixation nif3-like Nitrogen fixation protein Nif 3-like 97.06%

gltP Glutamate/aspartate: protein symporter gltP 98.72%

Nitrogen metabolism gltX Glutamate-tRNA ligase 98.97%

glnR Transcriptional repressor glnR 99.26%

glnA Type I glutamate- ammonia ligase/glutamine 98.73%

Nitrogen regulation nadR Transcription repressor nadR 98.15%

dissimulator nitrate nirB Nitrate reductase (NADH) large subunit 97.71%

nirD Nitrate reductase (NADH) small subunit 99.07%

nasD 97.60%

narl Respiratory nitrate reductase subunit gamma 98.85%

narH Nitrate reductase subunit beta/nitrate reductase 97.47%

narJ Molybdenum cofactor assembly chaperone 96.94%

nark Nitrate transporter 96.86%

**Supplementary Table.07. Genes involve in iron transportation**

**Pathway Genes Product Identity**

fbpA Fur-regulated basic protein fbpA 99.45%

fetB Iron export ABC transporter permease subunit fetB 97.48%

**Iron(III) transport** feuA Ferrichrome ABC transporter permease 81.73%

feuB FerrichromeABC transporter permease 81.23%

feuC Iron-uptake system permease feuC 97.23%

fecD Iron complex transport system permease protein 99.40%

**Supplementary Table.08. Genes involved in phosphate solubilization and transport**

**Pathway Genes Product Identity**

ispH 4-hydroxy-3-methylbut-2-enyl 97.88%

diphosphate reductase

pstA phosphate ABC transporter, permease protein PstA 97.63%

pstB phosphate import ATP-binding protein pstB 98.27%

pstC phosphate ABC transporter permease subunit PstC 98.82%

Degradation of pstS phosphate transport system permease protein 85.19%

Phosphonates gltP proton glutamate symport protein 98.72%

phoH phosphate starvation protein PhoH 99.38%

**Supplementary Table.09. Genes involved in sulfur metabolites**

**Pathway Genes Product Identity**

cysC adenylyl-sulfate kinase 98.65%

sat sulfate adenylyltransferase 97.91%

Sulfate transport cysK cysteine synthase A 98.17%

cysS cysteine--tRNA ligase 99.21%

sulP sulfate permease 97.64%

**Supplementary Table. 10. Genes involved in the production of volatiles organic compounds (VOCs)**

**Pathway Genes Product Identity**

acoA Acetion dehydrogenase 90.14%

acoB Acetion dehydrogenase 88.16%

Volatiles acoR Acetion dehydrogenase 82.61%

acuB Encoding acetoin 98.14%

acuC Encoding acetoin 98.72%

**Supplementary Table. 11. Comparison of genes related to plant growth promotion and volatiles genes between HAB-5, SRCM101359, FZB42, HAB-2, DSM7 and 168**

| Genes | Product | HAB-5 | SRCM101359 | FZB42 | HAB-2 | DSM7 | 168 |
| --- | --- | --- | --- | --- | --- | --- | --- |
| TrpA | Tryptophan synthase subunit alpha | **Yes** | **Yes** | **Yes** | **Yes** | **Yes** | **Yes** |
| TrpB | Tryptophan synthase subunit alpha | **Yes** | **Yes** | **Yes** | **Yes** | **Yes** | **Yes** |
| TrpC | Indole-3-glycerol phosphate synthase trpC | **Yes** | **Yes** | **Yes** | **Yes** | **Yes** | **Yes** |
| TrpD | Anthranilate phosphoribosyltransferase | **Yes** | **Yes** | **Yes** | **Yes** | **Yes** | **Yes** |
| TrpE | Anthranilate synthase component I | **Yes** | **Yes** | **Yes** | **Yes** | **Yes** | **Yes** |
| TrpS | Tryptophan-tRNA ligase | **Yes** | **Yes** | **Yes** | **Yes** | **Yes** | **Yes** |
| YwkB | Auxin efflux carrie | **Yes** | **Yes** | **No** | **No** | **No** | **Yes** |
| MiaA | Adenosinde-37-n6-dimethylallyltransferase | **Yes** | **Yes** | **Yes** | **Yes** | **Yes** | **Yes** |
| NadE | Ammonia-dependent NAD (+) synthase | **Yes** | **Yes** | **Yes** | **Yes** | **Yes** | **Yes** |
| FbpA | Fur-regulated basic protein | **Yes** | **Yes** | **Yes** | **Yes** | **Yes** | **Yes** |
| FetB | Iron export ABC transporter permease subunit | **Yes** | **Yes** | **Yes** | **Yes** | **Yes** | **Yes** |
| FeuC | Iron-uptake system permease | **Yes** | **Yes** | **Yes** | **Yes** | **Yes** | **Yes** |
| FeuB | ferrichromeABC transporter permease | **Yes** | **Yes** | **No** | **No** | **No** | **Yes** |
| FeuA | ferrichrome ABC transporter permease | **Yes** | **Yes** | **Yes** | **Yes** | **Yes** | **Yes** |
| nif3-like | Nitrogen fixation protein Nif 3-like | **Yes** | **Yes** | **Yes** | **Yes** | **Yes** | **Yes** |
| gltP | Glutamate/aspartate: protein symporter gltP | **Yes** | **Yes** | **Yes** | **Yes** | **Yes** | **Yes** |
| gltX | Glutamate-tRNA ligase | **Yes** | **Yes** | **Yes** | **Yes** | **Yes** | **Yes** |
| glnR | Transcriptional repressor | **Yes** | **Yes** | **Yes** | **Yes** | **Yes** | **Yes** |
| glnA | Type I glutamate- ammonia ligase/glutamine | **Yes** | **Yes** | **Yes** | **Yes** | **Yes** | **Yes** |
| nadR | Transcription repressor | **Yes** | **Yes** | **Yes** | **Yes** | **Yes** | **Yes** |
| nirB | Nitrate reductase (NADH) large subunit | **Yes** | **Yes** | **Yes** | **Yes** | **Yes** | **Yes** |
| nirD | Nitrate reductase (NADH) small subunit | **Yes** | **Yes** | **Yes** | **Yes** | **Yes** | **Yes** |
| nasD | assimilatory nitrite reductase subunit | **Yes** | **Yes** | **Yes** | **Yes** | **Yes** | **Yes** |
| narl | Respiratory nitrate reductase subunit gamma | **Yes** | **Yes** | **Yes** | **Yes** | **Yes** | **Yes** |
| narH | Nitrate reductase subunit beta/nitrate reductase | **Yes** | **Yes** | **Yes** | **Yes** | **Yes** | **Yes** |
| narJ | Molybdenum cofactor assembly chaperone | **Yes** | **Yes** | **Yes** | **Yes** | **Yes** | **Yes** |
| nark | Nitrate transporter | **Yes** | **Yes** | **Yes** | **Yes** | **Yes** | **Yes** |

**Supplementary Table. 12. comparative genes analysis of volatile chemicals between HAB-5, SRCM101359, FZB42, HAB-2, DSM7 and 168**

| Genes | Product | HAB-5 | SRCM101359 | FZB42 | HAB-2 | DSM7 | 168 |
| --- | --- | --- | --- | --- | --- | --- | --- |
| acoA | **Acetion dehyrogenase** | **Yes** | **Yes** | **No** | **No** | **No** | **Yes** |
| acoB | **Acetion dehyrogenase** | **Yes** | **Yes** | **No** | **No** | **No** | **Yes** |
| acoC | **Acetion dehyrogenase** | **No** | **No** | **No** | **No** | **No** | **Yes** |
| acoR | **Acetion dehyrogenase** | **Yes** | **Yes** | **No** | **No** | **No** | **Yes** |
| acuA | **acetion** | **No** | **No** | **No** | **No** | **Yes** | **No** |
| acuB | **acetion** | **Yes** | **Yes** | **No** | **No** | **No** | **No** |
| acuC | **acetion** | **Yes** | **Yes** | **No** | **No** | **Yes** | **No** |
| alsD | **acetolactate decarboxylase** | **No** | **No** | **Yes** | **Yes** | **No** | **No** |
| alsS | **acetolactate synthase** | **No** | **No** | **Yes** | **Yes** | **No** | **Yes** |
| alsR | **transcriptional regulator** | **No** | **No** | **Yes** | **Yes** | **No** | **No** |
| bdh | **2,3-butanediol dehydrogenase** | **No** | **No** | **Yes** | **Yes** | **No** | **No** |
| budA | **acetolatate decarboxylase** | **No** | **No** | **No** | **No** | **No** | **Yes** |
| butA | **2,3-butanediol** | **No** | **No** | **No** | **No** | **Yes** | **No** |
| butC | **2,3-butanediol** | **No** | **No** | **No** | **No** | **Yes** | **No** |


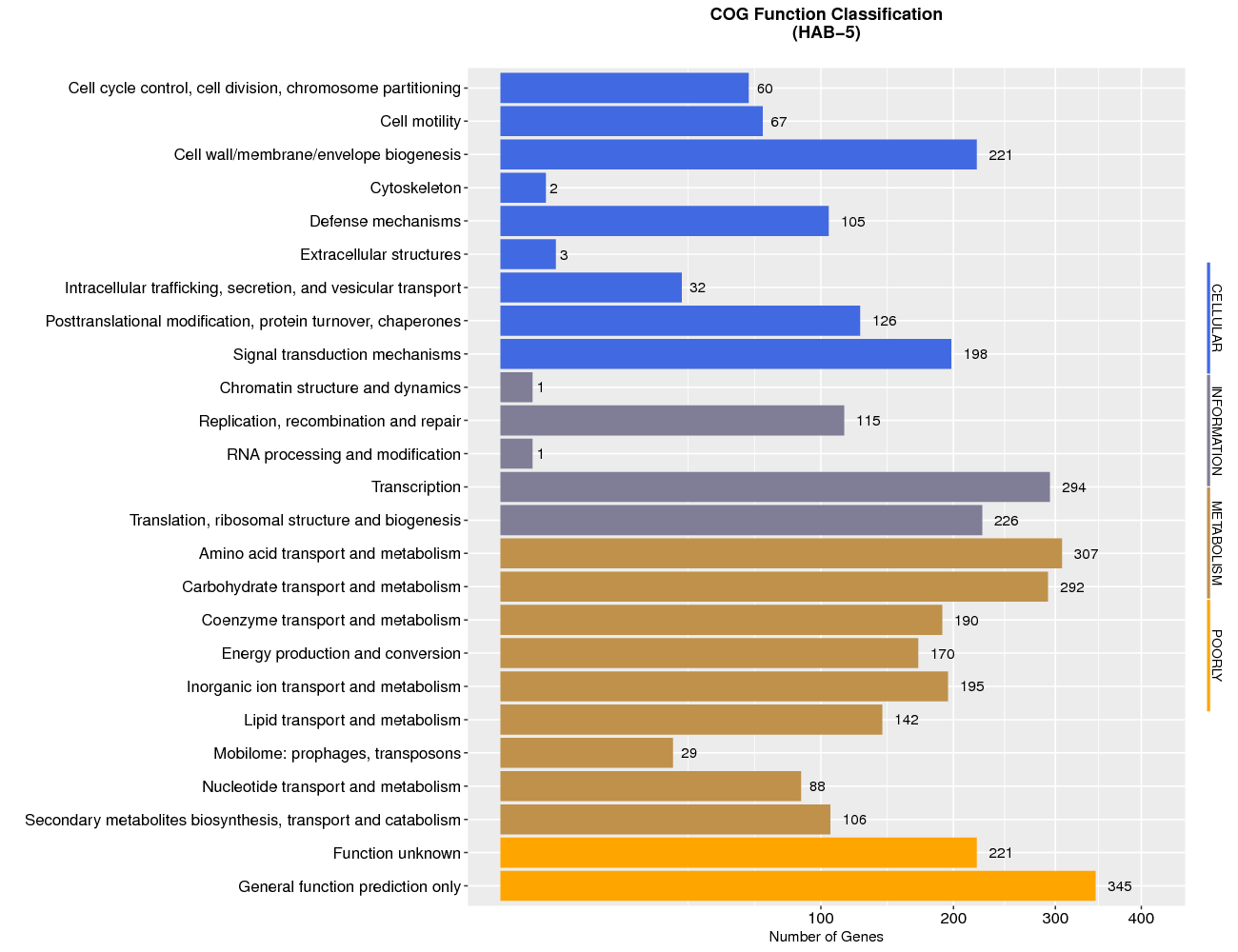


**SupplementaryFigure.02. Cluster of Orthologous Groups (COGS), Annotations of *B. atrophaeus* HAB-5 genes are categorized in four groups (Metabolism, Cellular Processes, Information and Poorly)**


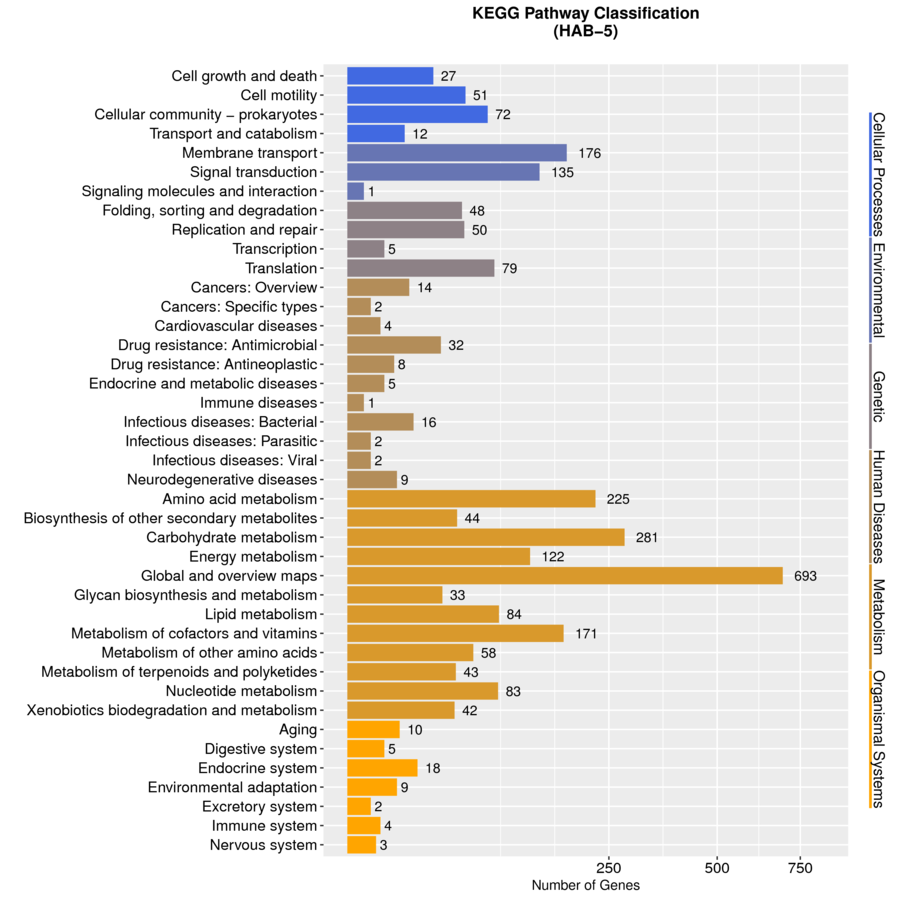


**Supplementary Figure.03. Kyoto Encyclopedia of Genes and Genomes (KEGG) analysis of *B. atrophaeus* HAB-5 genes are categorized in six groups (Cellular Processes, Environmental, Genetic, Human disease, Metabolism and Organismal systems)**


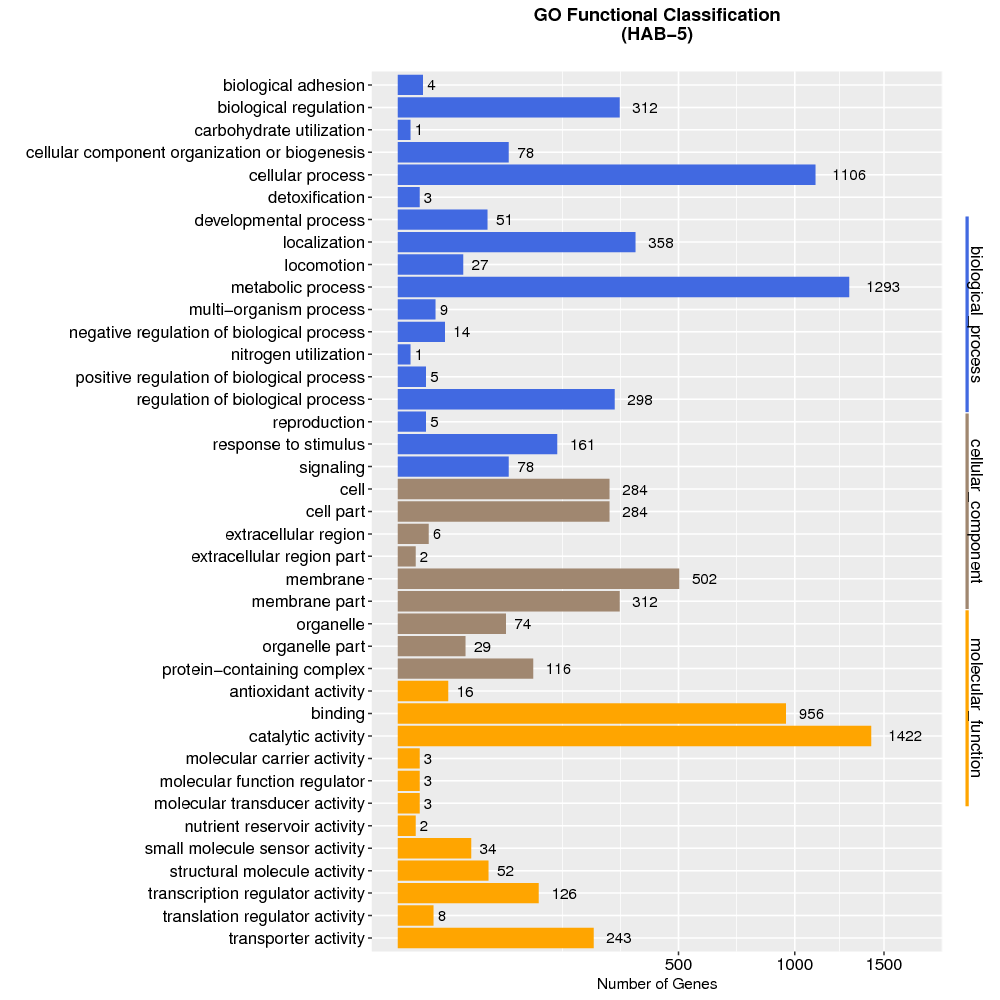


**Supplementary Figure.04. Gene Ontology (GO) analysis of *B. atrophaeus* HAB-5 genes are categorized in three groups (Biological Processes, Cellular Component and Molecular Functions )**


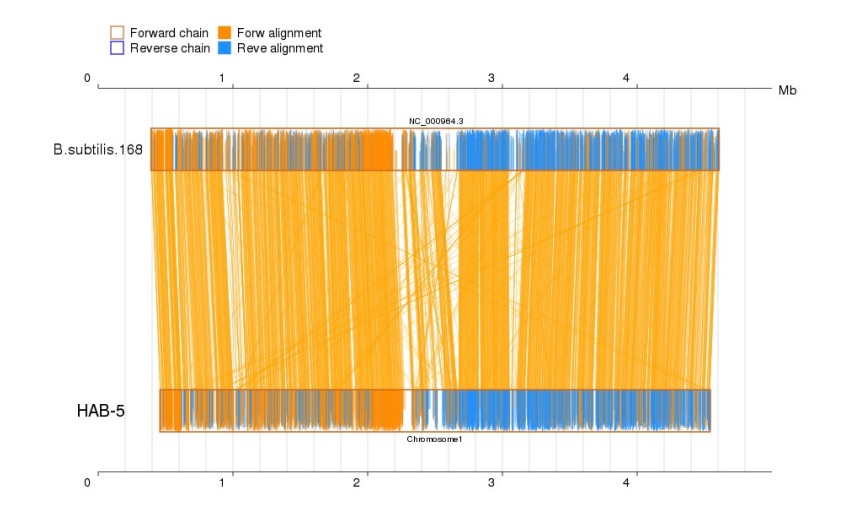


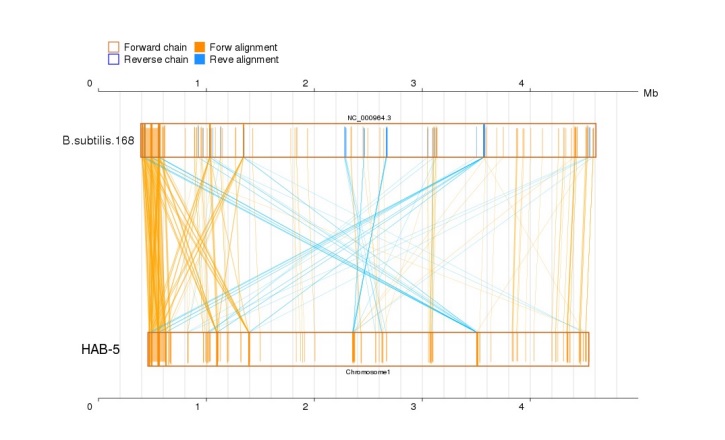


D

C


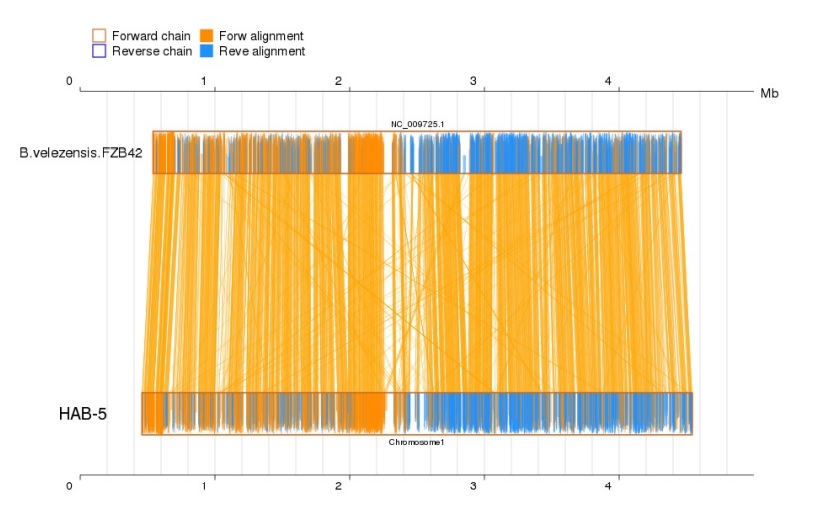

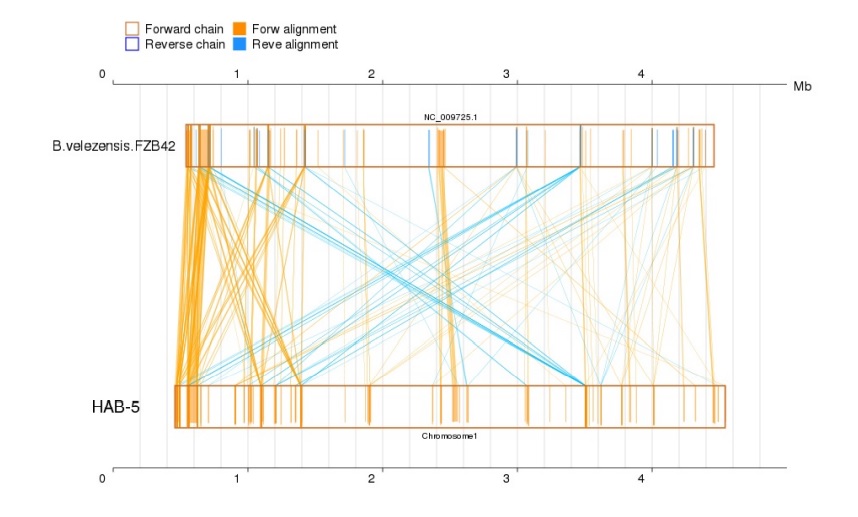


F

E


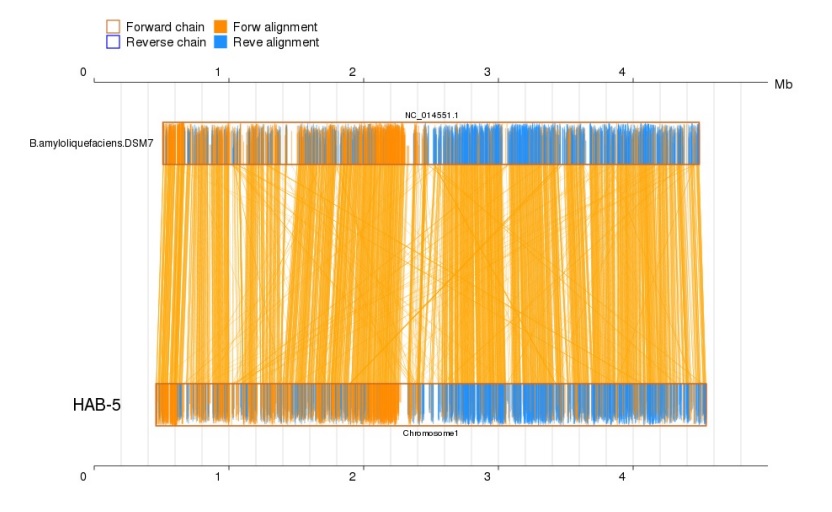

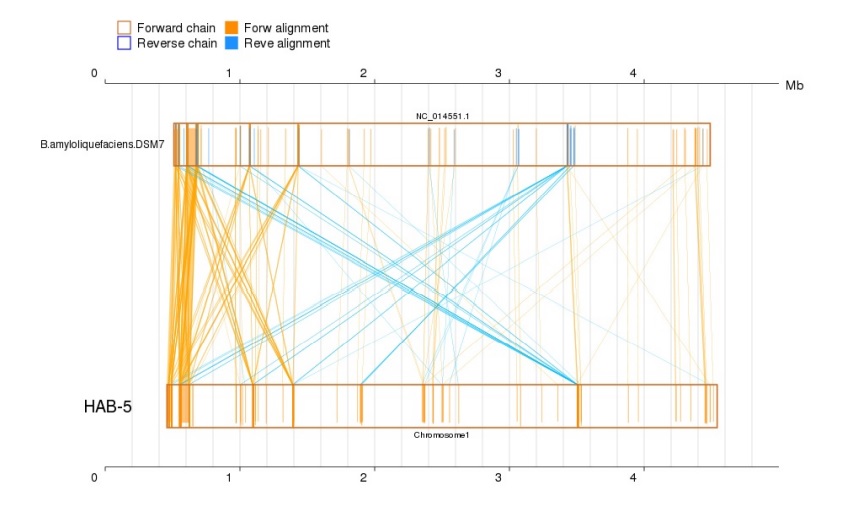


G


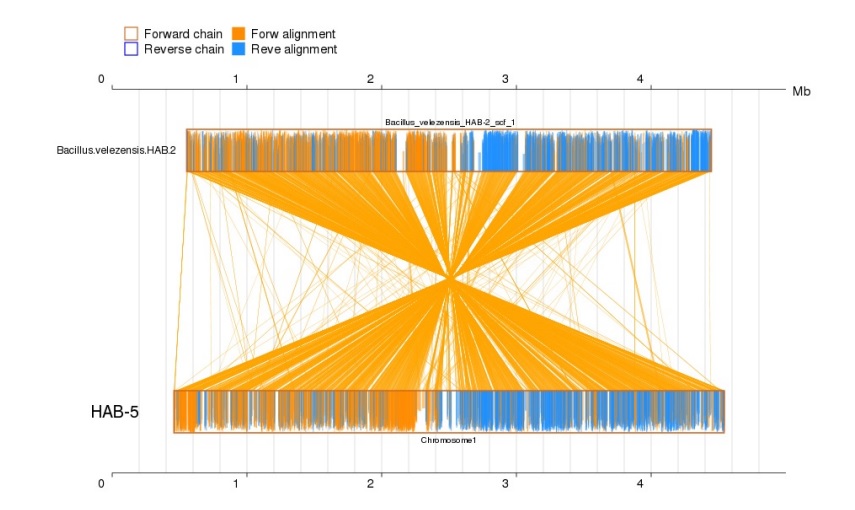

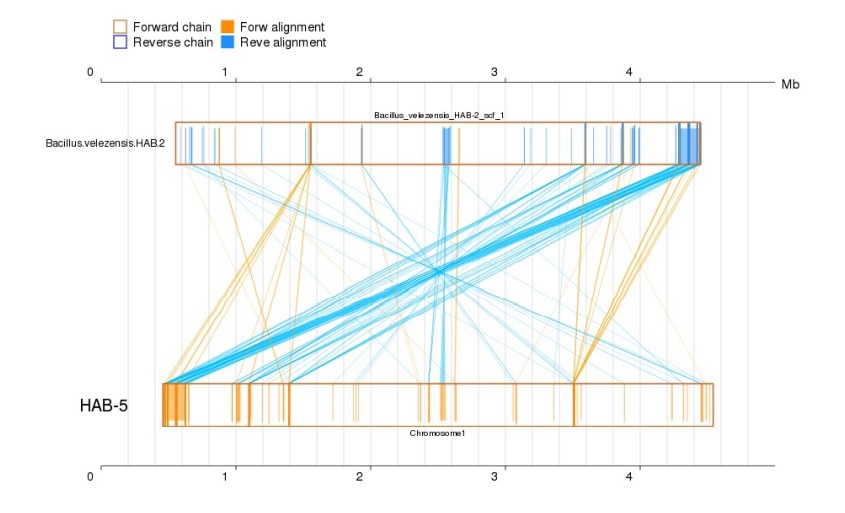


H

J

I


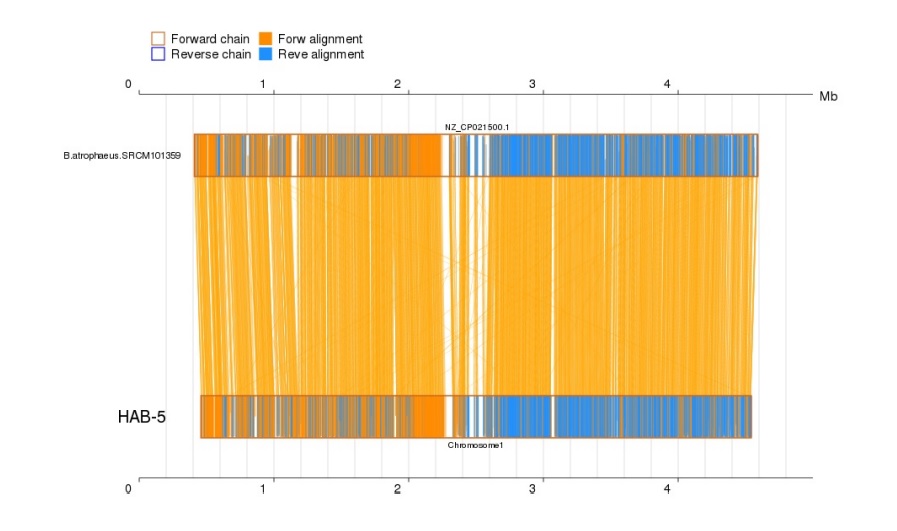

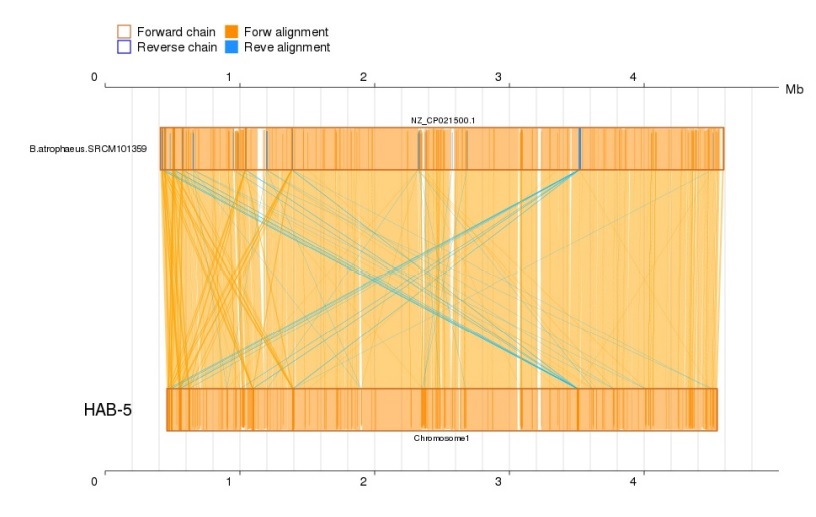


**SupplementaryFigure.08. The synteny of *Bacillus atrophaeus* HAB-5 with other *Bacillus* strains compared at nucleic acid level synteny and amino acid level synteny (A) *Bacillus atrophaeus* HAB-5 with *B. subtilis* 168 nucleic acid level synteny (B) *B. atrophaeus* HAB-5, *B. subtilis* 168 Amino acid level synteny (C) HAB-5 with *B.velezensis*.FZB42 nucleic acid level synteny (D) HAB-5 with *B. velezensis* FZB42 amino acid level synteny (E) HAB-5 with *B. amyloliquefaciens* DSM7 nucleic acid level synteny (F) HAB-5 with *B. amyloliquefaciens* DSM7 amino acid level synteny (G) HAB-5 with *B. velezensis* HAB.2 nucleic acid level synteny (H) HAB-5 with *B. velezensis* HAB.2 amino acid level synteny (I) HAB-5 with *B. atrophaeus* SRCM101359 nucleic acid level synteny (J) HAB-5 with *B. atrophaeus* SRCM101359 amino acid level synteny.**
